# Supplementary material for: Adaptive modeling and inference of higher-order coordination in neuronal assemblies: A dynamic greedy estimation approach
Source: PLoS Comput Biol. 2024 May 28;20(5):e1011605. doi: 10.1371/journal.pcbi.1011605 (PMC11161120; doi:10.1371/journal.pcbi.1011605)
Supplement: S1 Appendix — In this appendix, we present supporting information regarding algorithm development and our theoretical results supporting the proposed statistical inference framework. (PDF) [file pcbi.1011605.s001.pdf]

# S1 Appendix
# Algorithms, Derivations, and Theoretical Results for "Adaptive modeling and inference of higher-order coordination in neuronal assemblies: a dynamic greedy estimation approach"


Shoutik Mukherjee[1,2], Behtash Babadi[1,2*]

**1** Department of Electrical and Computer Engineering, University of Maryland, College Park, Maryland, United States of America
**2** Institute for Systems Research, University of Maryland, College Park, Maryland, United States of America

* behtash@umd.edu


**Overview.** In this appendix, we present supporting information regarding algorithm development and our theoretical results supporting the proposed statistical inference framework. Sections A–C address the details of the problem formulation and algorithm development. In Section A, the discretized marked point process model (MkPP) and its adaptive estimation are formulated, both in the history-dependent and history-independent cases. In Section B, the estimation of both the history-dependent (Section B.1) and history-independent (Section B.2) discretized marked point process (MkPP) models are addressed. Section C describes the filtering/smoothing algorithm applied to estimated exogenous factors, $\{\hat{\gamma}_k^{(m)}\}_{k=1:K}^{m\in\mathcal{K}_r}$, used to quantify the null hypothesis in tests of $r^{\text{th}}$-order coordination.

Sections D–E describe the theoretical results supporting our proposed statistical inference framework, with proofs provided in F. In order to precisely characterize the limiting distribution of our test statistic, the adaptive de-biased deviance difference, we first establish that confidence intervals can be constructed around greedily estimated parameters in Section D. We show that the de-sparsification technique proposed in [1] applies to the AdOMP estimate (Section D.1), and that the de-sparsified AdOMP estimate asymptotically behaves like the maximum likelihood estimate D.2. Then, we use this result to characterize the limiting behavior of the adaptive de-biased deviance difference in Section E. We address the main result for testing $r^{\text{th}}$-order coordination using history-dependent models in Section E.1. Next, we address the special case of history-independent models in corollary (Section E.2). In a second corollary result, we consider the general case of testing nested hypotheses with history-dependent models (Section E.3).

# A Problem Formulation

This section provides detailed formulations of the MkPP model and the adaptive estimation problem to complement their overviews in the main text.

## A.1 Discretized marked point process likelihood model

Because multivariate point processes, as defined in literature [2], do not permit simultaneous events at arbitrarily small time scales, point process models of ensemble spiking treat neurons as conditionally independent elements of a multivariate process. To avoid this assumption, Solo introduced a theoretical model for simultaneous spiking events based on MkPPs that explicitly model each possible ensemble spiking event [3]. Due to the convenience of its disjoint decomposition, MkPP representations have been used by Kass et al. in [4] and Ba et al. in [5] to model simultaneous spiking in neuronal assemblies.

We utilize a discrete-time MkPP representation of ensemble neuronal activity [5]. For an assembly of $C$ neurons, the $C$-variate spiking process, binned with small bin size $\Delta$, at time bin index $t$ is denoted by $\boldsymbol{n}_t := [n_t^{(1)}, n_t^{(2)}, \ldots, n_t^{(C)}]^\top$, where each component is the spiking process of one neuron. Conventional discrete point process models treat the components as conditionally independent Bernoulli observations. Given our interest in simultaneous spikes, we instead treat $\boldsymbol{n}_t$ as multivariate Bernoulli observations. The spiking process $\boldsymbol{n}_t$ is mapped to a $C^*$-variate process $\boldsymbol{n}_t^* := [n_t^{*(1)}, n_t^{*(2)}, \ldots, n_t^{*(C^*)}]^\top$, which are the binned observations of a marked point process whose marks count the number of exactly one of $C^* := 2^C - 1$ disjoint non-zero spiking events; we refer to $\boldsymbol{n}_t^*$ as the *marked* Bernoulli process, distinguishing it from the multivariate Bernoulli process $\boldsymbol{n}_t$. We define the mark space $\mathcal{K} := \{1, \ldots, C^*\}$ [2]. At each time $t_j$ such that $\boldsymbol{n}_{t_j} \neq \boldsymbol{0}$, the sole non-zero element of $\boldsymbol{n}_{t_j}^*$ indicates the mark. We also define the binned ground process $n_t^{(g)}$ that takes value 1 at each such $t_j$ and is zero otherwise [2]; the ground process indicates the occurrence of any spiking event and is represented by $n_t^{(g)} := \sum_{m=1}^{C^*} n_t^{*(m)}$.

The marked process representation is not uniquely defined, but can be done so in a convenient fashion: treating the components of $\boldsymbol{n}_t$ as the bits of a $C$-bit binary number, the mark indexed by the decimal equivalent of a particular realization of $\boldsymbol{n}_t$ will corresponded to that realization. By the disjointness of the marked representation, the spiking process of the $c^{\text{th}}$ neuron can be recovered as the sum of all marked processes whose index, in binary, takes value 1 at the $c^{\text{th}}$ bit. The conditional intensity functions (CIFs) of $\boldsymbol{n}_t$ and $\boldsymbol{n}_t^*$ are approximated by the probabilities of observing an event at time bin $t$ given ensemble spiking history. That is,

$$\lambda_t^{(c)}\Delta = \mathbb{P}[n_t^{(c)} = 1|\mathcal{H}_t], \qquad \lambda_t^{*(m)}\Delta = \mathbb{P}[n_t^{*(m)} = 1|\mathcal{H}_t], \tag{1}$$

for $c = 1, \ldots, C$ and $m = 1, \ldots, C^*$. We can relate $\lambda_t^{(c)}\Delta$ to $\lambda_t^{*(m)}\Delta$ in the same manner as $n_t^{(c)}$ to $n_t^{*(m)}$, and obtain the CIF of the ground process $\lambda_t^{*(g)}\Delta = \sum_{m=1}^{C^*} \lambda_t^{*(m)}\Delta$.

The marked process permits a generative description of simultaneous spiking events: ensemble spiking events are characterized by the ground process, occurring with probability $\lambda_t^{*(g)}\Delta$; the event is then assigned to the $m^{\text{th}}$ mark (i.e. the $m^{\text{th}}$ simultaneous spiking outcome) with conditional probability $\frac{\lambda_t^{*(m)}\Delta}{\lambda_t^{*(g)}\Delta}$. Thus, at time $t$ the likelihood of ensemble event $\boldsymbol{n}_t^*$ is given by:

$$p(\boldsymbol{n}_t^*) = \prod_{m=1}^{C^*} \left(\frac{\lambda_t^{*(m)}\Delta}{\lambda_t^{*(g)}\Delta}\right)^{n_t^{*(m)}} \left(\lambda_t^{*(g)}\Delta\right)^{n_t^{(g)}} \left(1 - \lambda_t^{*(g)}\Delta\right)^{1-n_t^{(g)}}. \tag{2}$$

The likelihood in Eq. (2) is used to form a multinomial generalized linear model (mGLM) with multinomial logistic link function of which we consider two versions.

The first, more general version utilizes the ensemble history as covariates in the mGLM. Letting the covariate vector $\boldsymbol{x}_t$ be the ensemble history up to some fixed lag at time $t$ (augmented by a constant element of 1), the model is parameterized by $\boldsymbol{\omega}_t := [\boldsymbol{\omega}_t^{(1)^\top}, \boldsymbol{\omega}_t^{(2)^\top}, \ldots, \boldsymbol{\omega}_t^{(C^*)^\top}]^\top$, where the parameters for the $m^{\mathsf{th}}$ mark $\boldsymbol{\omega}_t^{(m)} := [\mu_t^{(m)}, \boldsymbol{\theta}_t^{(m)^\top}]^\top$ consists of an ensemble history-modulation vector $\boldsymbol{\theta}_t^{(m)}$ and the baseline firing parameter, $\mu_t^{(m)}$. The CIF of the $m^{\mathsf{th}}$ mark is hence defined as:

$$\lambda_t^{*(m)}\Delta := \frac{e^{\boldsymbol{x}_t^\top \boldsymbol{\omega}_t^{(m)}}}{1 + \sum_{j=1}^{C^*} e^{\boldsymbol{x}_t^\top \boldsymbol{\omega}_t^{(j)}}}, \quad m = 1, 2, \cdots, C^*; \tag{3}$$

equivalently, the log-odds of the $m^{\mathsf{th}}$ mark occurring versus no spiking event occurring are $\boldsymbol{x}_t^\top \boldsymbol{\omega}_t^{(m)} = \log\left(\frac{\lambda_t^{*(m)}\Delta}{1 - \lambda_t^{*(g)}\Delta}\right)$.

The second version of the mGLM makes the simplifying assumption that there is no history dependence. The resulting model depends only on contemporaneous spiking, permitting compact parameterization by the baseline firing parameters $\boldsymbol{\mu}_t = [\mu_t^{(1)}, \mu_t^{(2)}, \ldots, \mu_t^{(C^*)}]^\top$. In the history-independent model, the log-odds of the $m^{\mathsf{th}}$ mark occurring versus no spiking event occurring are:

$$\mu_t^{(m)} = \log\left(\frac{\lambda_t^{*(m)}\Delta}{1 - \lambda_t^{*(g)}\Delta}\right). \tag{4}$$

The log-likelihood can be expressed in a fashion resembling the maximum entropy model [6, 7]. For the history-independent case, the log-likelihood of $\boldsymbol{n}_t^*$ is

$$\log p(\boldsymbol{n}_t^*) = \boldsymbol{\mu}_t^\top \boldsymbol{n}_t^* - \psi(\boldsymbol{\mu}_t), \quad \text{where} \quad \psi(\boldsymbol{\mu}_t) := \log\left(1 + \sum_{m=1}^{C^*} e^{\mu_t^{(m)}}\right), \tag{5}$$

while the history-dependent formulation admits a similar expression by simply replacing $\mu_t^{(m)}$ with $\boldsymbol{x}_t^\top \boldsymbol{\omega}_t^{(m)}$.

## A.2 Adaptive estimation of marked point process models

First, recall that since parameters of the MkPP mGLM are allowed to change in time, we use adaptive algorithms to capture the dynamics of the history-dependent and history-independent models; and that for tractabilitiy, we employ a thresholding rule similar to [8] thus considering only "reliable interactions", i.e. the subset of the mark space $\bar{\mathcal{K}} = \{m \in \mathcal{K} : \sum_t n_t^{*(m)} > N_{thr}\}$ for some pre-defined constant $N_{thr} > 0$. For generality and clarity in notation, the following derivations are in terms of the full mark space $\mathcal{K}$.

The parameters of the history-dependent discretized MkPP model are adaptively estimated by solving a sequence of maximum likelihood problems. We assume that the parameters $\boldsymbol{\omega}_t$ admit piece-wise constant dynamics and are constant over consecutive windows of length $W$; additionally, observations are assumed conditionally independent across time bins. The ensemble history up to lag $p$ defines the covariates as $\boldsymbol{x}_t := [1, n_{t-1}^{(1)}, \ldots, n_{t-p}^{(1)}, \ldots, n_{t-1}^{(C)}, \ldots, n_{t-p}^{(C)}]^\top$. The set of history covariate vectors at the $i^{\mathsf{th}}$ window are denoted by $\boldsymbol{X}_i = [\boldsymbol{x}_{1+i(W-1)}, \ldots, \boldsymbol{x}_{iW}]^\top$. Note that since the mapping from $\boldsymbol{n}_t^*$ to $\boldsymbol{n}_t$ is injective, the influence of past spiking activity can be equivalently captured by defining history covariates in terms of either; however, using $\boldsymbol{n}_t$ reduces the dimensionality of $\boldsymbol{\omega}_t$ and quantifies the influence of past spiking activity directly rather than through categorical variables. Let $\boldsymbol{n}_i^{*(m)} = [n_{1+W(i-1)}^{*(m)}, \ldots, n_{iW}^{*(m)}]^\top$

denote the sequence of outcomes of the $m^{\text{th}}$ mark in the $i^{\text{th}}$ window. The log-likelihood of the $i^{\text{th}}$ window is thus given by

$$\ell_i(\boldsymbol{\omega}_i) := \sum_{m=1}^{C^*} \boldsymbol{n}_i^{*(m)^\top} \boldsymbol{X}_i \boldsymbol{\omega}_i^{(m)} - \sum_{j=1+(i-1)W}^{iW} \log \left(1 + \sum_{m=1}^{C^*} e^{\boldsymbol{x}_j{}^\top \boldsymbol{\omega}_i^{(m)}}\right). \tag{6}$$

Motivated by the RLS objective function [9], a forgetting factor mechanism is utilized to combine the log-likelihoods up to the $k^{\text{th}}$ window, capturing the dynamics in each mark's rates. For a forgetting factor $0 \leq \beta < 1$, the adaptively-weighted log-likelihood at window $k$ is thus defined as:

$$\ell_k^\beta(\boldsymbol{\omega}_k) := (1-\beta) \sum_{i=1}^{k} \beta^{k-i} \ell_i(\boldsymbol{\omega}_k). \tag{7}$$

Parameter estimation is hence performed by solving the sequence of maximum likelihood problems:

$$\hat{\boldsymbol{\omega}}_k := \underset{\boldsymbol{\omega}_k}{\arg\max} \quad \ell_k^\beta(\boldsymbol{\omega}_k), \quad k = 1, 2, \cdots, K. \tag{8}$$

To efficiently solve the sequence of problems in Eq. (8) in an online fashion, we use the Adaptive Orthogonal Matching Pursuit (AdOMP) [10], an adaptive version of the Orthogonal Matching Pursuit (OMP) [11] [12]. The AdOMP and OMP both iteratively identify the parameter support set (the non-zero components) of fixed size (a hyperparameter for which we cross-validate) to encourage sparsity, thus capturing the inherent sparsity of network interactions based on past ensemble activity [13–15]. However, AdOMP permits the support set to change between windows. Moreover, greedy estimation over a sparse subset of parameters mitigates the intractability of the estimation problem for large neuronal assemblies where regularization-based constraints still require optimization over all parameters. The AdOMP relies on efficient evaluation of the gradient $\nabla_{\boldsymbol{\omega}} \ell_k^\beta(\boldsymbol{\omega}_k)$ at the $l^{\text{th}}$ iterate $\hat{\boldsymbol{\omega}}_{(l),k}$, to determine the next addition to the parameter support set and to solve the new maximization problem via gradient descent. Hence, its recursive computation is crucial for the algorithm to operate in an online fashion. To this end, we utilize a recursive update rule to compute the gradient at the $k^{\text{th}}$ window, generalizing the adaptive filtering techniques employed in [16] for Bernoulli observations to a multivariate setting. A complete derivation of the recursive update rule and the utilization of the gradient in AdOMP is provided in Section B.1.

The sequence of maximum likelihood problems that must be solved to obtain the history-independent model takes a similar form as in Eqs. (6) – (8) under the same assumption of piece-wise constant dynamics of $\boldsymbol{\mu}_t$. Defining $\bar{\boldsymbol{n}}_i^* := \frac{1}{W} \sum_{j=(i-1)W+1}^{iW} \boldsymbol{n}_j^*$, the equivalent of the $i^{\text{th}}$ window log-likelihood in Eq. (6) is given by $\ell_i(\boldsymbol{\mu}_i) = W(\boldsymbol{\mu}_i^\top \bar{\boldsymbol{n}}_i^* - \psi(\boldsymbol{\mu}_i))$. Hence, the adaptively-weighted log-likelihood at the $k^{\text{th}}$ window is:

$$\begin{aligned} \ell_k^\beta(\boldsymbol{\mu}_k) &:= (1-\beta) \sum_{i=1}^{k} \beta^{k-i} \ell_i(\boldsymbol{\mu}_k) \\ &= (1-\beta) \sum_{i=1}^{k} W \beta^{k-i} (\boldsymbol{\mu}_k^\top \bar{\boldsymbol{n}}_i^* - \psi(\boldsymbol{\mu}_k)). \end{aligned} \tag{9}$$

The sequence of maximum likelihood estimates

$$\hat{\boldsymbol{\mu}}_k = \underset{\boldsymbol{\mu}_k}{\arg\max} \quad \ell_k^\beta(\boldsymbol{\mu}_k), \quad k = 1, 2, \cdots, K \tag{10}$$

are obtained by gradient descent. The gradient of the history-independent log-likelihood in Eq. (9), while involving a weighted summation of the terms $\bar{\boldsymbol{n}}_i^*$ for which a simple recursion is derived, can be computed directly. The procedure for computing the maximum-likelihood estimates of the history-independent model is detailed in Section B.2.

## A.3 Statistical inference of higher-order coordination

Here, we expound on the formulation of a nested hypothesis test for $r^{\text{th}}$-order coordination of ensemble spiking activity presented in the main text. Recall that the significance of $r$-wise simultaneous spiking for $r \geq 2$ is tested by considering the two alternatives:

$$\begin{aligned} H_0 &: r^{\text{th}}\text{-order simultaneous spikes occur as frequently as they would between independent units, given ensemble spiking history} \\ H_1 &: r^{\text{th}}\text{-order simultaneous spikes occur at a significantly different rate than they would between independent units, given ensemble spiking history.} \end{aligned} \tag{11}$$

We estimate a *reduced* model that assumes $r^{\text{th}}$-order interactions are chance occurrences by constraining the base rate parameters for each $r^{\text{th}}$-order mark. For the $m^{\text{th}}$ mark, let

$$u_t^{(m)} := \boldsymbol{x}_t{}^\top \boldsymbol{\omega}_k^{(m)} = \mu_k^{(m)} + \bar{\boldsymbol{x}}_t^\top \boldsymbol{\theta}_k^{(m)}. \tag{12}$$

We decompose the base rate parameter as

$$\mu_k^{(m)} = \mu_{0,k}^{(m)} + \gamma_k^{(m)}, \tag{13}$$

where $\mu_{0,k}^{(m)}$ is the base rate under the null hypothesis and $\gamma_k^{(m)}$ is analogous to the additional multiplicative factor in [4] that captures potential exogenous effects after conditioning on ensemble spiking history.

The *reduced* model is estimated by solving the sequence of maximum likelihood problems $\hat{\boldsymbol{\omega}}_k^{(R)} := \arg\max_{\boldsymbol{\omega}_k^{(R)}} \ \ell_k^\beta(\boldsymbol{\omega}_k^{(R)})$ with the base rate parameters of $r^{\text{th}}$-order events constrained to their values under the null hypothesis. Let

$$\mathcal{K}_r := \left\{ m \in \mathcal{K} : \sum_{c=1}^{C} m_c = r \right\}, \tag{14}$$

where $m_c$ is the $c^{\text{th}}$ least significant bit of $m$ in binary. Then, to obtain the *reduced* model, we fix $\mu_k^{(m)}$ to $\mu_{0,k}^{(m)}$ for each $m \in \mathcal{K}_r$ and optimize the remaining parameters. To explicitly obtain the constraints, first recall that $u_t^{(m)}$ is the log-odds of $n_t^{*(m)} = 1$ versus $n_t^{(g)} = 0$ given ensemble spiking history. Under the assumption that the neurons are independent, the probabilities of each event are given, respectively, by

$$\begin{cases} \mathbb{P}[n_t^{*(m)} = 1 | \mathcal{H}_t] = \displaystyle\prod_{c_a : m_{c_a} = 1} \left( \lambda_t^{(c_a)} \Delta \right) \prod_{c_b : m_{c_b} = 0} \left( 1 - \lambda_t^{(c_b)} \Delta \right), \text{ and} \\ \mathbb{P}[n_t^{(g)} = 0 | \mathcal{H}_t] = \displaystyle\prod_{c=1}^{C} \left( 1 - \lambda_t^{(c)} \Delta \right). \end{cases} \tag{15}$$

Evaluating the log-ratio of these two probabilities at the full model estimate $\hat{\boldsymbol{\omega}}_k$, we obtain that

$$u_{0,t}^{(m)} := \sum_{c : m_c = 1} \log \left( \frac{\hat{\lambda}_t^{(c)} \Delta}{1 - \hat{\lambda}_t^{(c)} \Delta} \right). \tag{16}$$

Attributing the difference between $u_t^{(m)}$ and $u_{0,t}^{(m)}$ only to exogenous factors, we estimate the exogenous factors at the $k^{\text{th}}$ window as

$$\hat{\gamma}_k^{(m)} = \frac{1}{W} \sum_{t=(k-1)W+1}^{kW} \big(u_t^{(m)} - u_{0,t}^{(m)}\big). \tag{17}$$

Thus, for the reduced model, the value of $\mu_k^{(m)}$ is constrained to $\hat{\mu}_k^{(m)} - \hat{\gamma}_k^{(m)}$ for $m \in \mathcal{K}_r$. The hypotheses are then quantitatively stated as:

$$H_0 : \boldsymbol{\omega}_k = \hat{\boldsymbol{\omega}}_k^{(R)}, \qquad H_1 : \boldsymbol{\omega}_k \neq \hat{\boldsymbol{\omega}}_k^{(R)}. \tag{18}$$

To control the possible abrupt variations of $\hat{\gamma}_k^{(m)}$ across windows, we apply Kalman forward/backward smoothing to the exogenous factor and use the smoothed values, $\tilde{\gamma}_k^{(m)}$, in lieu of $\hat{\gamma}_k^{(m)}$. The procedure is detailed in Section C.

# B Estimating MkPP Models

This section states the Adaptive Orthogonal Matching Pursuit (AdOMP) algorithm proposed in [10] and provides the derivations of recursive update rules for the log-likelihood and its gradient, which are utilized to efficiently implement AdOMP and computate the adaptive de-biased deviance difference (Section B.1). The section also restates the recursive algorithm for maximum likelihood estimation of the history-independent model (Section B.2), proposed in [17].

## B.1 The History-Dependent Model: Recursive Gradient Computation and AdOMP

We use AdOMP [10] to greedily estimate the parameters of the history-dependent MkPP model to capture the inherent sparsity of network interactions [13–15] and for tractability. The algorithm uses the gradient of the log-likelihood at each iteration of the matching pursuit to determine the next addition to the parameter support set and to solve the new maximization problem with a gradient descent algorithm. We derive a recursive update to compute the gradient at the $k^{\text{th}}$ window.

The gradient of the objective subsumes the gradients with respect to each $\boldsymbol{\omega}^{(m)}$. That is, $\nabla_{\boldsymbol{\omega}} \ell_k^{\beta}(\boldsymbol{\omega}_k) = \left[\nabla_{\boldsymbol{\omega}^{(1)}} \ell_k^{\beta}(\boldsymbol{\omega}_k), \ldots, \nabla_{\boldsymbol{\omega}^{(C^*)}} \ell_k^{\beta}(\boldsymbol{\omega}_k)\right]^{\top}$, where, for each $m = 1, \ldots, C^*$,

$$\nabla_{\boldsymbol{\omega}^{(m)}} \ell_k^{\beta}(\boldsymbol{\omega}_k) = \sum_{i=1}^{k} \beta^{k-i} \nabla_{\boldsymbol{\omega}^{(m)}} \ell_i(\boldsymbol{\omega}_k) = \sum_{i=1}^{k} \beta^{k-i} \boldsymbol{X}_i^{\top} \left[\boldsymbol{n}_i^{*(m)} - \boldsymbol{\lambda}_i^{*(m)}(\boldsymbol{\omega}_k)\Delta\right]. \quad (19)$$

The $m^{\text{th}}$ mark's CIF over the $i^{\text{th}}$ window is denoted by $\boldsymbol{\lambda}_i^{*(m)}(\boldsymbol{\omega}_k)\Delta = [\lambda^*_{1+(i-1)W}{}^{(m)}(\boldsymbol{\omega}_k)\Delta, \ldots, \lambda^*_{iW}{}^{(m)}(\boldsymbol{\omega}_k)\Delta]^{\top}$, and the common term $(1-\beta)$ is dropped for convenience, as it does not affect the optimal solution. Observe that the gradient, evaluated at the $l^{\text{th}}$ iteration $\hat{\boldsymbol{\omega}}_{(l),k}$, can equivalently be written as

$$\nabla_{\boldsymbol{\omega}} \ell_k^{\beta}(\hat{\boldsymbol{\omega}}_{(l),k}) = \beta \left(\nabla_{\boldsymbol{\omega}} \ell_{k-1}^{\beta}(\hat{\boldsymbol{\omega}}_{(l),k})\right) + \nabla_{\boldsymbol{\omega}} \ell_k(\hat{\boldsymbol{\omega}}_{(l),k}), \quad (20)$$

suggesting a recursive update. However, this definition requires the value of the gradient (and consequentially, the CIFs) from the previous window evaluated at $\hat{\boldsymbol{\omega}}_{(l),k}$, which is unavailable in an online setting. However, the values $\boldsymbol{\lambda}_i^*(\hat{\boldsymbol{\omega}}_i)\Delta$ have been evaluated at previous windows; thus, each $\boldsymbol{\lambda}_i^*(\hat{\boldsymbol{\omega}}_{(l),k})\Delta$ is approximated by its first-order Taylor approximation around $\hat{\boldsymbol{\omega}}_i$. In the following, we consider only the $m^{\text{th}}$ mark, noting that each component of the gradient may be obtained by applying the procedure to each mark in parallel.

First, the aforementioned Taylor approximation is computed for the CIFs of the $m^{\text{th}}$ mark at window $i^{\text{th}}$. Defining the diagonal matrix

$$\boldsymbol{\Lambda}_i^{*(m,j)} := \begin{cases} diag\left(\boldsymbol{\lambda}_i^{*(m)}\Delta \odot (1 - \boldsymbol{\lambda}_i^{*(j)}\Delta)\right), & m = j \\ \\ diag\left(\boldsymbol{\lambda}_i^{*(m)}\Delta \odot \boldsymbol{\lambda}_i^{*(j)}\Delta\right), & m \neq j \end{cases} \quad (21)$$

with diagonal terms given by the specified point-wise products, we find the first-order Taylor approximation to be

$$\boldsymbol{\lambda}_i^{*(m)}(\hat{\boldsymbol{\omega}}_{(l),k})\Delta \approx \boldsymbol{\lambda}_i^{*(m)}(\hat{\boldsymbol{\omega}}_i)\Delta + \sum_{j=1}^{C^*} \boldsymbol{\Lambda}_i^{*(m,j)}(\hat{\boldsymbol{\omega}}_i)\ \boldsymbol{X}_i \left(\hat{\boldsymbol{\omega}}_{(l),k}^{(j)} - \hat{\boldsymbol{\omega}}_i^{(j)}\right). \quad (22)$$

Note that evaluating the approximation of the CIF for the $m^{\text{th}}$ requires the CIFs of the other marks through $\boldsymbol{\Lambda}_i^{*(m,j)}$. Making the simplifying assumption that $(\lambda_i^{*(m)}\Delta) \cdot (\lambda_i^{*(j)}\Delta) = o(\Delta)$, $m \neq j$, the cross-terms are rendered negligible and the parallelized CIF approximation is given by

$$\boldsymbol{\lambda}^{*}_{(l),k}{}^{(m)}\Delta := \boldsymbol{\lambda}_i^{*(m)}(\hat{\boldsymbol{\omega}}_{(l),k})\Delta \approx \boldsymbol{\lambda}_i^{*(m)}(\hat{\boldsymbol{\omega}}_i)\Delta + \boldsymbol{\Lambda}_i^{*(m,m)}(\hat{\boldsymbol{\omega}}_i)\ \boldsymbol{X}_i \left(\hat{\boldsymbol{\omega}}_{(l),k}^{(m)} - \hat{\boldsymbol{\omega}}_i^{(m)}\right). \quad (23)$$

Substituting this approximation, the gradient evaluated at $\hat{\boldsymbol{\omega}}_{(l),k}$ becomes

$$\boldsymbol{g}_{(l),k}^{(m)} := \nabla_{\boldsymbol{\omega}^{(m)}} \ell_k^{\beta}(\hat{\boldsymbol{\omega}}_{(l),k}) \approx \sum_{i=1}^{k} \beta^{k-i} \left[ \boldsymbol{X}_i{}^{\top} \boldsymbol{\varepsilon}_{(l),i}^{(m)} - \boldsymbol{X}_i{}^{\top} \boldsymbol{\Lambda}_i^{*(m,m)} \boldsymbol{X}_i \left( \hat{\boldsymbol{\omega}}_{(l),k}^{(m)} - \hat{\boldsymbol{\omega}}_i^{(m)} \right) \right], \quad (24)$$

where $\boldsymbol{\varepsilon}_{(l),i}^{(m)} := \boldsymbol{n}_i^{*(m)} - \boldsymbol{\lambda}^{*}_{(l),i}{}^{(m)}\Delta$. Defining the quantities

$$\begin{aligned} \boldsymbol{b}_k^{(m)} &:= \beta \boldsymbol{b}_{k-1}^{(m)} + \boldsymbol{X}_k{}^{\top} \boldsymbol{\varepsilon}_{(l),k}^{(m)} + \boldsymbol{X}_k{}^{\top} \boldsymbol{\Lambda}_k^{*(m,m)}(\hat{\boldsymbol{\omega}}_{(l),k}) \boldsymbol{X}_k \hat{\boldsymbol{\omega}}_{(l),k}^{(m)}, \\ \boldsymbol{B}_k^{(m)} &:= \beta \boldsymbol{B}_{k-1}^{(m)} + \boldsymbol{X}_k{}^{\top} \boldsymbol{\Lambda}_k^{*(m,m)}(\hat{\boldsymbol{\omega}}_{(l),k}) \boldsymbol{X}_k, \end{aligned} \quad (25)$$

the parallelized, fully recursive gradient update rule for the $m^{\text{th}}$ mark is thus

$$\begin{aligned} \boldsymbol{g}_{(l),k}^{(m)} =& \boldsymbol{b}_k^{(m)} - \boldsymbol{B}_k^{(m)} \hat{\boldsymbol{\omega}}_{(l),k}^{(m)} \\ =& \beta \left( \boldsymbol{b}_{k-1}^{(m)} - \boldsymbol{B}_{k-1}^{(m)} \hat{\boldsymbol{\omega}}_{(l),k}^{(m)} \right) + \boldsymbol{X}_k{}^{\top} \boldsymbol{\varepsilon}_{(l),k}^{(m)}. \end{aligned} \quad (26)$$

The AdOMP algorithm, and the utility of the recursive gradient update, is detailed in Algorithm A. Let the initial support set of size $r_0$ be denoted by $S^{(0)}$, and the maximum support size by $s^*$. The maximum support size can be obtained by cross-validation. We assume that $S^{(0)}$ is non-empty here, where a reasonable initialization is made; it is then necessary to solve the preliminary problem in Step 3. Alternatively, we may initially set $S^{(0)} = \emptyset$ and $\hat{\boldsymbol{\omega}}_{(0),k} = \mathbf{0}$.

Additionally, the log-likelihoods of the full and reduced models can be computed in an online fashion. We use the Taylor approximation of $\ell_i(\hat{\boldsymbol{\omega}}_k)$ around $\hat{\boldsymbol{\omega}}_i$ to obtain the following recursion:

$$\ell_k^{\beta}(\hat{\boldsymbol{\omega}}_k) = a_k + \sum_{m=1}^{C^*} \left( \hat{\boldsymbol{\omega}}_k^{(m)\top} \boldsymbol{b}_k^{(m)} - \frac{1}{2} \hat{\boldsymbol{\omega}}_k^{(m)\top} \boldsymbol{B}_k^{(m)} \hat{\boldsymbol{\omega}}_k^{(m)} \right), \quad (27)$$

where the variable $a_k$ is defined as

$$a_k := \beta a_{k-1} + \ell_k(\hat{\boldsymbol{\omega}}_k) - \sum_{m=1}^{C^*} \left( \hat{\boldsymbol{\omega}}_k^{(m)\top} \boldsymbol{X}_k^{\top} \boldsymbol{\varepsilon}_k^{(m)} + \frac{1}{2} \hat{\boldsymbol{\omega}}_k^{(m)\top} \boldsymbol{X}_k^{\top} \boldsymbol{\Lambda}^{*(m)}_k \boldsymbol{X}_k \hat{\boldsymbol{\omega}}_k^{(m)} \right), \quad (28)$$

and $\boldsymbol{b}_k$, $\boldsymbol{B}_k$ have been previously defined. The bias term $\mathscr{B}_k = \left(\nabla \ell_k^{\beta}(\hat{\boldsymbol{\omega}}_k)\right)^{\top} \left(\nabla^2 \ell_k^{\beta}(\hat{\boldsymbol{\omega}}_k)\right)^{-1} \left(\nabla \ell_k^{\beta}(\hat{\boldsymbol{\omega}}_k)\right)$ can also be computed recursively. The recursive computations of the log-likelihood and bias terms enable the adaptive de-biased deviance difference to be computed efficiently.

## B.2 The History-Independent Model: Maximum Likelihood Estimation

The maximum likelihood estimates of the history-independent discretized MkPP model's parameters are obtained via by gradient descent; Algorithm B, below, details

**Algorithm A** AdOMP : **Ad**aptive **O**rthogonal **M**atching **P**ursuit

**Input:** $\{\boldsymbol{n}_k^{*(m)}\}_{m=1}^{C^*}$, $\boldsymbol{X}_k$, $\{\boldsymbol{b}_{k-1}^{(m)}\}_{m=1}^{C^*}$, $\{\boldsymbol{B}_{k-1}^{(m)}\}_{m=1}^{C^*}$, $S^{(0)}$, $s^*$, $\beta$
**Output:** $\hat{\boldsymbol{\omega}}_k$, $\{\boldsymbol{b}_k^{(m)}\}_{m=1}^{C^*}$, $\{\boldsymbol{B}_k^{(m)}\}_{m=1}^{C^*}$

$r_0 = |S^{(0)}|$
$S_k^{(r_0)} = S^{(0)}$
$\hat{\boldsymbol{\omega}}_{(r_0),k} = \arg\max_{\text{supp}(\boldsymbol{\omega}_k)\subseteq S_k^{(r_0)}} \ell_k^\beta(\boldsymbol{\omega}_k)$
**for** $r = r_0 + 1$ to $s^*$ **do**
  **for** $m = 1$ to $C^*$ **do**
    $\boldsymbol{g}_{(r),k}^{(m)} = \beta \boldsymbol{g}_{(r-1),k-1}^{(m)} + {\boldsymbol{X}_k}^\top \boldsymbol{\varepsilon}_{(r-1),k}^{(m)}$
  **end for**
  $j = \arg\max_{i\notin S_k^{(r)}} \left|\{g_{(r),k}^{(i)}\}\right|$
  $S_k^{(r)} = S_k^{(r-1)} \cup \{j\}$
  $\hat{\boldsymbol{\omega}}_{(r),k} = \arg\max_{\text{supp}(\boldsymbol{\omega}_k)\subseteq S_k^{(r)}} \ell_k^\beta(\boldsymbol{\omega}_k)$
**end for**
**for** $m = 1$ to $C^*$ **do**
  ${\boldsymbol{\Lambda}_{(s^*),k}^*}^{(m)} = diag\left({\boldsymbol{\lambda}_{(s^*),k}^*}^{(m)}\Delta \odot (1 - {\boldsymbol{\lambda}_{(s^*),k}^*}^{(m)}\Delta)\right)$
  $\boldsymbol{b}_k^{(m)} = \beta \boldsymbol{b}_{k-1}^{(m)} + {\boldsymbol{X}_k}^\top \boldsymbol{\varepsilon}_{(s^*),k}^{(m)} + {\boldsymbol{X}_k}^\top {\boldsymbol{\Lambda}_{(s^*),k}^*}^{(m)} \boldsymbol{X}_k \hat{\boldsymbol{\omega}}_{(s^*),k}^{(m)}$
  $\boldsymbol{B}_k^{(m)} = \beta \boldsymbol{B}_{k-1}^{(m)} + {\boldsymbol{X}_k}^\top {\boldsymbol{\Lambda}_{(s^*),k}^*}^{(m)} \boldsymbol{X}_k$
**end for**
**return** $\hat{\boldsymbol{\omega}}_k = \hat{\boldsymbol{\omega}}_{(s^*),k}$, $\{\boldsymbol{b}_k^{(m)}\}_{m=1}^{C^*}$, $\{\boldsymbol{B}_k^{(m)}\}_{m=1}^{C^*}$

the implementation proposed in [17] and utilized here. Noting that the gradient at window $k$ involves the weighted summation of $\bar{\boldsymbol{n}}_i^*$, a simple recursion can be used to compute it efficiently at each window. The log-likelihoods of the full and reduced history-independent models are similarly straightforward to compute using the same recursive variables. The parameters $I_{max}$ and $\kappa$ are the maximum number of gradient-descent iterations and step size, respectively. In step 4, the term $\exp(\hat{\boldsymbol{\mu}}_k)$ is understood to be an element-wise operation.

**Algorithm B** ML Estimation of History-Independent Model

**Input:** $\{\bar{\boldsymbol{n}}_k^*\}_{k=1}^K$, $\beta$, $I_{max}$, $\kappa$
**Output:** $\{\hat{\boldsymbol{\mu}}_k\}_{k=1}^K$
  *Initialization:* $\boldsymbol{x}_0 = \mathbf{0}$, $\{\hat{\boldsymbol{\mu}}_k\}_{k=1}^K = \mathbf{0}$
1: **for** $k = 1$ to $K$ **do**
2:   $\boldsymbol{x}_k = \beta \boldsymbol{x}_{k-1} + \bar{\boldsymbol{n}}_k^*$
3:   **for** $iter = 1$ to $I_{max}$ **do**
4:     $\hat{\boldsymbol{\lambda}}_k^*\Delta \leftarrow \exp(\hat{\boldsymbol{\mu}}_k) / \left(1 + \sum_{m\in\mathcal{K}} \exp(\hat{\mu}_k^{(m)})\right)$
5:     $\nabla \ell_k^\beta(\hat{\boldsymbol{\mu}}_k) = W\left(\boldsymbol{x}_k - \frac{1-\beta^k}{1-\beta}\hat{\boldsymbol{\lambda}}_k^*\Delta\right)$
6:     $\hat{\boldsymbol{\mu}}_k \leftarrow \hat{\boldsymbol{\mu}}_k + \kappa\, \nabla \ell_k^\beta(\hat{\boldsymbol{\mu}}_k)$
7:   **end for**
8: **end for**
9: **return** $\{\hat{\boldsymbol{\mu}}_k\}_{k=1}^K$

# C Forward-Backward Smoothing of Exogenous Factors

In our proposed test for $r^{\mathsf{th}}$-order coordination, the null hypothesis that $r^{\mathsf{th}}$-order spiking occurs at the same rate as between $r$ independent neurons is quantified by estimating a reduced model for which $\mu_k^{(m)}$ is fixed at $\hat{\mu}_k^{(m)} - \hat{\gamma}_k^{(m)}$ for $m \in \mathcal{K}_r$, where the estimated exogenous factors $\hat{\gamma}_k^{(m)} = \frac{1}{W} \sum_{t=(k-1)W+1}^{kW} (u_t^{(m)} - u_{0,t}^{(m)})$ as stated in the main text. However, despite parameters being piece-wise constant, mark CIFs in the history-dependent model (hence $u_t^{(m)}$ and $u_{0,t}^{(m)}$) can vary abruptly between adjacent time-bins due ensemble spiking history covariates; in turn, this propagates to the estimated exogenous factors. Hence, we apply Kalman forward/backward smoothing [18] for stability in the estimated exogenous factors over time.

The procedure is summarized in Algorithm C for the estimated exogenous factors of the $m^{\mathsf{th}}$ mark, $\{\hat{\gamma}_k^{(m)}\}_{k=1}^K$. The forward-backward smoothing algorithm was applied independently for each $m \in \mathcal{K}_r$. The model is stated in Eq. (29), below:

$$\begin{aligned} \hat{\gamma}_k &= x_k + v_k, & v_k &\sim \mathcal{N}(0, \sigma_v^2), \\ x_k &= \rho x_{k-1} + w_k, & w_k &\sim \mathcal{N}(0, \sigma_w^2). \end{aligned} \tag{29}$$

Dropping the mark index from the notation for clarity, we assume that $\{\hat{\gamma}_k\}_{k=1}^K$ are the noisy observations of a linear Gaussian model with state transition coefficient $\rho \in (0, 1]$. Noise samples, $v_k$ and $w_k$, are independent and normally distributed, with respective variances $\sigma_v^2$ and $\sigma_w^2$.

**Algorithm C** Smoothing $\{\hat{\gamma}_k\}_{k=1}^K$ via Kalman Forward/Backward Algorithm

**Input:** $\{\hat{\gamma}_k\}_{k=1}^K$, $\sigma^2_{v,(0)}$, $\sigma^2_{w,(0)}$, $\rho$, $L$
**Output:** $\{\breve{\gamma}_k\}_{k=1}^K$, $\{\tilde{\gamma}_k\}_{k=1}^K$, $\sigma^2_{v,(L)}$, $\sigma^2_{w,(L)}$

1: **for** $l = 1$ to $L$ **do**
2: $\quad \sigma^2_{0|0} = 1$, $x_{0|0} = 0$
3: $\quad$ **for** $k = 1$ to $K$ **do**
4: $\quad\quad x_{k|k-1} = \rho x_{k-1|k-1}$
5: $\quad\quad \sigma^2_{k|k-1} = \rho^2 \sigma^2_{k-1|k-1} + \sigma^2_{w,(l-1)}$
6: $\quad\quad x_{k|k} = x_{k|k-1} + \frac{\sigma^2_{k|k-1}}{\sigma^2_{k|k-1} + \sigma^2_{v,(l-1)}} \left(\hat{\gamma}_k - x_{k|k-1}\right)$
7: $\quad\quad \sigma^2_{k|k} = \sigma^2_{k|k-1} \frac{\sigma^2_{v,(l-1)}}{\sigma^2_{k|k-1} + \sigma^2_{v,(l-1)}}$
8: $\quad$ **end for**
9: $\quad$ **for** $k = K$ to $1$ **do**
10: $\quad\quad x_{k-1|K} = x_{k-1|k-1} + \frac{\sigma^2_{k-1|k-1}}{\sigma^2_{k|k-1}} \left(x_{k|K} - x_{k|k-1}\right)$
11: $\quad\quad \sigma^2_{k-1|K} = \sigma^2_{k-1|k-1} + \left(\frac{\sigma^2_{k-1|k-1}}{\sigma^2_{k|k-1}}\right)^2 \left(\sigma^2_{k|K} - \sigma^2_{k|k-1}\right)$
12: $\quad$ **end for**
13: $\quad \sigma^2_{w,(l)} = \frac{1}{K} \sum_{k=1}^K \left[\left(\hat{\gamma}_k - x_{k|K}\right)^2 + \sigma^2_{k|K}\right]$
14: $\quad \sigma^2_{w,(l)} = \frac{1}{K} \sum_{k=1}^K \left[\left(x_{k|K} - x_{k-1|K}\right)^2 + \left(1 - 2\frac{\sigma^2_{k-1|k-1}}{\sigma^2_{k|k-1}}\right) \sigma^2_{k|K} + \sigma^2_{k-1|K}\right]$
15: **end for**
16: $\{\breve{\gamma}_k\}_{k=1}^K = \{x_{k|k}\}_{k=1}^K$ and $\{\tilde{\gamma}_k\}_{k=1}^K = \{x_{k|K}\}_{k=1}^K$
17: **return** $\{\breve{\gamma}_k\}_{k=1}^K$, $\{\tilde{\gamma}_k\}_{k=1}^K$, $\sigma^2_{v,(L)}$, $\sigma^2_{w,(L)}$

Steps 3–8 describe the forward filter, while backward smoothing is described in Steps 9–12. Additionally, an Expectation-Maximization algorithm is used to update the values

of the noise variances $\sigma_w^2$ and $\sigma_v^2$ over $L$ iterations. In simulations, we observed that in $L = 10$ iterations, noise variances converged to stable solutions after being initialized to $\sigma^2_{w,(0)} = \sigma^2_{v,(0)} = 10 \times \frac{1}{2}\text{Var}(\{\hat{\gamma}_k\}_{k=1:K})$; this initialization scheme and choice of $L$ was also used in real data applications. The backward-smoothed estimated exogenous factors, $\{\tilde{\gamma}_k\}_{k=1}^K$, were utilized to compute null values of the baseline rate parameters.

# D Asymptotic Normality of the De-Sparsified AdOMP Estimate

Precisely characterizing the limiting behavior of the adaptive de-biased deviance, our test statistic, requires the ability to construct confidence intervals around parameter estimates. While procedures are well-established for unrestricted or unregularized linear regression models, there is a notable gap in existing literature on greedily-estimated high-dimensional sparse models. However, a set of elegant results [1,19,20] in relation to $\ell_1$-regularized maximum-likelihood estimation have established techniques to de-sparsify parameter estimates and construct confidence intervals. In this section, we extend the de-sparsification technique of [1] to the greedy high-dimensional setting (Section D.1) and prove the de-sparsified AdOMP estimate also asymptotically behaves like the maximum likelihood estimate (Section D.2).

## D.1 De-Sparsifying the AdOMP Estimate

We show that the AdOMP parameter estimates can be de-sparsified as in [1] by inspecting the Karush-Kuhn-Tucker (KKT) conditions of the optimization problem. First, note from Algorithm A that $\hat{\boldsymbol{\omega}}_k = \hat{\boldsymbol{\omega}}_{(s^*),k}$, solves the maximization problem

$$\hat{\boldsymbol{\omega}}_{(s^*),k} = \underset{\mathrm{supp}(\boldsymbol{\omega}_k)\subseteq S_k^{(s^*)}}{\arg\max} \ \ell_k^{\beta}(\boldsymbol{\omega}_k). \tag{30}$$

The support constraint is equivalent to requiring that $\omega_k^{(i)} = 0, i \notin S_k^{(s^*)}$. Collectively, these linear equality constraints can be expressed as $A_k^{(s^*)}\boldsymbol{\omega}_k = \mathbf{0}$, where the diagonal matrix $\left[A_k^{(s^*)}\right]_{i,i} = 0$ for $i \in S_k^{(s^*)}$ and 1 otherwise. The KKT conditions on the primal and dual parameters ($\boldsymbol{\omega}_k$ and $\boldsymbol{\nu}_k^{(s^*)}$, respectively) are straightforward to derive. Of particular relevance is the stationarity condition:

$$\nabla\ell_k^{\beta}(\boldsymbol{\omega}_k) - A_k^{(s^*)}\boldsymbol{\nu}_k^{(s^*)} = \mathbf{0}. \tag{31}$$

Substituting for $\ell_k^{\beta}(\boldsymbol{\omega}_k)$ with its quadratic approximation around $\hat{\boldsymbol{\omega}}_{(s^*),k}$ in the stationarity condition and rearranging terms, the de-sparsified parameters are defined as

$$\hat{\boldsymbol{w}}_k := \hat{\boldsymbol{\omega}}_{(s^*),k} - \left(\nabla^2\ell_k^{\beta}(\hat{\boldsymbol{\omega}}_{(s^*),k})\right)^{-1}\left(\nabla\ell_k^{\beta}(\hat{\boldsymbol{\omega}}_{(s^*),k})\right), \tag{32}$$

in the same fashion as van de Geer et al. in [1].

## D.2 Asymptotic Normality

Next, we establish the asymptotic normality of the de-sparsified AdOMP estimate. While a similar result was established in [1], the independence of each realization of the covariates and observations was assumed; additionally, several technical conditions were tailored for $\ell_1$-regularized maximum likelihood estimation. Hence, the result is formally stated based on conditions revised for our setting, enumerated below.

(C1) For $i = 1, \ldots, k$, define $\boldsymbol{z}_i := [\boldsymbol{z}_i^{(1)\top}, \ldots, \boldsymbol{z}_i^{(C^*)\top}]^\top$ such that $\{\boldsymbol{z}_i^{(m)}\}_{m=1:C^*} := \{\boldsymbol{X}_i\boldsymbol{\omega}_k^{(m)}\}_{m=1:C^*}$. Writing $\ell_i(\boldsymbol{\omega}_k)$ equivalently as $\ell_i(\{\boldsymbol{z}_i^{(m)}\}_{m=1:C^*})$, the second derivatives $\nabla^2_{\boldsymbol{z}_i}\ell_i(\{\boldsymbol{z}_i^{(m)}\}_{m=1:C^*})$ exist and satisfy the following for all $\boldsymbol{z}_i$ and for some $\delta$-neighborhood ($\delta > 0$):

$$\max_{\boldsymbol{z}_0\in\{\boldsymbol{z}_i\}_{i=1}^k}\left\{\sup_{|\hat{\boldsymbol{z}}-\boldsymbol{z}_0|\vee|\boldsymbol{z}-\boldsymbol{z}_0|<\delta}\frac{\|\nabla^2_{\boldsymbol{z}_i}\ell_i(\{\hat{\boldsymbol{z}}_i^{(m)}\}_{m=1}^{C^*}) - \nabla^2_{\boldsymbol{z}_i}\ell_i(\{\boldsymbol{z}_i^{(m)}\}_{m=1}^{C^*})\|}{\|\hat{\boldsymbol{z}}-\boldsymbol{z}\|}\right\} \leq 1$$

(C2) Assuming the true parameters, $\boldsymbol{\omega}_k \in \mathbb{R}^d$, to be $(s, \xi)$-compressible with $\xi < \frac{1}{2}$, as in [21], the error $\|\hat{\boldsymbol{\omega}}_k - \boldsymbol{\omega}_k^0\|_1 = \mathcal{O}_{\mathbb{P}}(\zeta\sqrt{d})$, where $\zeta := \sqrt{(1-\beta)\log(s)\log(d)}$ and $\boldsymbol{\omega}_k^0$ is the maximum likelihood estimate.

(C3) Letting $\hat{\Sigma}_k := \nabla^2 \ell_k^\beta(\hat{\boldsymbol{\omega}}_k)$, and $\Sigma_k := \mathbb{E}\left[\nabla^2 \ell_k(\boldsymbol{\omega}_k)\right]$, one of the following two conditions holds. For every $\beta_l$ in the monotonically increasing sequence $\{\beta_l\}_{l=1}^{\infty}$ that converges to 1, $\hat{\Theta}_k = \hat{\Sigma}_k^{-1}$ exists. Alternatively, $\hat{\Theta}_k$ is a consistent estimator of $\Sigma_k^{-1}$, and $\|\hat{\Theta}_k \hat{\Sigma}_k - \boldsymbol{I}\|_\infty = \mathcal{O}_{\mathbb{P}}(\sqrt{1-\beta})$.

(C4) The covariates satisfy $\|\boldsymbol{X}\|_\infty := \max \left|[\boldsymbol{X}]_{i,j}\right| = \mathcal{O}(c_1)$ for some constant $c_1$, where $\boldsymbol{X} := \left[\boldsymbol{X}_1{}^\top, \ldots, \boldsymbol{X}_k{}^\top\right]^\top$ is the collection of history covariate matrices over all windows.

(C5) The covariates satisfy $\|\tilde{\boldsymbol{X}} \hat{\Theta}_k\|_\infty = \mathcal{O}_{\mathbb{P}}(c_1)$, where the $T \times d$ matrix $\tilde{\boldsymbol{X}}$ is obtained by tiling $\boldsymbol{X}$ horizontally $C^*$ times.

(C6) The covariates satisfy $\|\tilde{\boldsymbol{X}}(\hat{\boldsymbol{\omega}}_k - \boldsymbol{\omega}_k^0)\|^2 = \mathcal{O}_{\mathbb{P}}\left(c_2 \zeta^2\right)$ for some constant $c_2$.

**Theorem 1.** *Consider the maximization of the total data log-likelihood $\ell_k^\beta(\boldsymbol{\omega}_k)$ at the $k^{\text{th}}$ window, where the true parameter $\boldsymbol{\omega}_k \in \mathbb{R}^d$ is $(s, \xi)$-compressible with $\xi < \frac{1}{2}$. Let $\boldsymbol{\omega}_k^0$ be the maximum likelihood estimate and $\hat{\boldsymbol{\omega}}_k$ be the AdOMP estimate after $\mathcal{O}(s log(s))$ iterations. Under conditions (C1)–(C6), the de-sparsified AdOMP estimate $\hat{\boldsymbol{w}}_k$ satisfies*

$$\sqrt{\frac{1+\beta}{1-\beta}}\left(\hat{\boldsymbol{w}}_k - \boldsymbol{\omega}_k^0\right) = \boldsymbol{V}_k + o_{\mathbb{P}}(1) \cdot \mathbf{1},$$

*where, as $\beta \to 1$, $\boldsymbol{V}_k \xrightarrow{d} \mathcal{N}(\mathbf{0}, \mathcal{I}_k^{-1})$ with $\mathcal{I}_k^{-1} = -\Sigma_k^{-1}$ the inverse of the Fisher information matrix.*

The complete proof is provided in Section F.2, but is summarized here. Theorem 1 is established by decomposing the difference $\left(\hat{\boldsymbol{w}}_k - \boldsymbol{\omega}_k^0\right)$ into two components, one of which is negligible, using conditions (C1)-(C6). The rate of decay of negligible terms is explicitly provided, but summarily treated as $o_{\mathbb{P}}(1)$. Then, modified versions of the Central Limit Theorem and Law of Large Numbers are used to show the non-negligible component is asymptotically normal. Based on Theorem 1, confidence intervals for the AdOMP estimates can be constructed by adapting the recursive procedure of [16] to our setting.

# E Limiting Behavior of the Adaptive De-biased Deviance Difference

In this section, we characterize the limiting distributions of the adaptive de-biased deviance difference under both the null and alternative hypotheses using the results in Section D. We first address the main result for testing $r^{\text{th}}$-order coordination using history-dependent models in Section E.1. Recalling that the history-independent models are special cases of the history-dependent models analyzed in our main result, we specifically address the former in a corollary result (Section E.2). Finally, in a second corollary result, we extend the main result to tests of arbitrary nested hypotheses (Section E.3).

## E.1 Testing for $r^{\text{th}}$-Order Coordination with History-Dependent Models

First, the limiting distributions of the adaptive de-biased deviance difference for history-dependent MkPP models are characterized under both the null hypothesis of chance $r^{\text{th}}$-order spiking and the alternative hypothesis of coordinated $r^{\text{th}}$-order spiking in Theorem 2, below.

**Theorem 2.** *Let $\hat{\boldsymbol{\omega}}_k^{(F)}$ and $\hat{\boldsymbol{\omega}}_k^{(R)}$ respectively be the full and reduced greedily-estimated parameters of the history-dependent model at window $k$, where $\hat{\boldsymbol{\omega}}_k^{(R)}$ assumes conditionally independent $r^{\text{th}}$-order simultaneous spiking. Then, as $\beta \to 1$,*

*i) if $r^{\text{th}}$-order coordination matches independent $r^{\text{th}}$-order interactions given ensemble spiking history, then $D_{k,\beta}^{(r)}\big(\hat{\boldsymbol{\omega}}_k^{(F)}, \hat{\boldsymbol{\omega}}_k^{(R)}\big) \xrightarrow{d} \chi^2(M^{(r)})$, i.e. chi-square, and*

*ii) if $r^{\text{th}}$-order coordination differs from independent $r^{\text{th}}$-order interactions given ensemble spiking history, and difference between the estimated base rate parameters of $r^{\text{th}}$-order interactions and their restricted values scales at least as $\mathcal{O}\Big(\sqrt{\frac{1-\beta}{1+\beta}}\Big)$, then $D_{k,\beta}^{(r)}\big(\hat{\boldsymbol{\omega}}_k^{(F)}, \hat{\boldsymbol{\omega}}_k^{(R)}\big) \xrightarrow{d} \chi^2(M^{(r)}, \nu_k^{(r)})$, i.e. non-central chi-square,*

*where $\nu_k^{(r)}$ is the non-centrality parameter at window $k$ and the degrees of freedom $M^{(r)} := |\mathcal{K}_r|$ is the difference in the cardinalities of the full and reduced support sets.*

The complete proof is presented in Section F.3. It is based on Davidson and Lever's treatment in [22] and is closely related to similar analyses for inferring adaptive Granger causality [23].

## E.2 Special Case: History-Independent Models

The statistical inference procedure described in the main text for the history-dependent MkPP model can be specialized for history-independent analysis, as has been previously demonstrated [17]. In this case, the two hypotheses,

$H_0$ : $r^{\text{th}}$-order simultaneous spikes occur as frequently as they would between independent units

$H_1$ : $r^{\text{th}}$-order simultaneous spikes occur at a significantly different rate than they would between independent units

(33)

are quantified in the same manner as the history-dependent case, excepting the history covariates. The distinction between the history-independent and the history-dependent test is that the former seeks only to determine if observed rates of simultaneous spiking

events are facilitated or suppressed, while the latter seeks to determine if observed rates of simultaneous spiking events are attributable to unobserved or neglected processes.

Estimates of the history-independent model parameters are unbiased, as no sparsity constraints are imposed. Hence, the adaptive deviance difference, $D_{k,\beta}^{(r)}\left(\hat{\boldsymbol{\mu}}_k^{(F)}, \hat{\boldsymbol{\mu}}_k^{(R)}\right) := \left(\frac{1+\beta}{1-\beta}\right)\left[2\left(\ell_k^{\beta}(\hat{\boldsymbol{\mu}}_k^{(F)}) - \ell_k^{\beta}(\hat{\boldsymbol{\mu}}_k^{(R)})\right)\right]$, is used as the test statistic. Here, we characterize the asymptotic distributions of the adaptive deviance difference under the null and alternative hypotheses in a corollary to Theorem 2.

**Corollary 2.1.** *Let $\hat{\boldsymbol{\mu}}_k^{(F)}$ and $\hat{\boldsymbol{\mu}}_k^{(R)}$ respectively be the full and reduced maximum-likelihood estimates of the history-independent model parameters at window $k$, where $\hat{\boldsymbol{\mu}}_k^{(R)}$ assumes conditionally independent $r^{\text{th}}$-order simultaneous spiking. Then, as $\beta \to 1$,*

*i) if $r^{\text{th}}$-order synchrony matches independent $r^{\text{th}}$-order interactions, the adaptive deviance difference $D_{k,\beta}^{(r)}\left(\hat{\boldsymbol{\mu}}_k^{(F)}, \hat{\boldsymbol{\mu}}_k^{(R)}\right) \xrightarrow{d} \chi^2(M^{(r)})$, and*

*ii) if $r^{\text{th}}$-order synchrony differs from independent $r^{\text{th}}$-order interactions, and the difference between the estimated base rate parameters of $r^{\text{th}}$-order interactions and their restricted values scales at least as $\mathcal{O}(\sqrt{\frac{1-\beta}{1+\beta}})$, the adaptive deviance difference $D_{k,\beta}^{(r)}\left(\hat{\boldsymbol{\mu}}_k^{(F)}, \hat{\boldsymbol{\mu}}_k^{(R)}\right) \xrightarrow{d} \chi^2(M^{(r)}, \nu_k^{(r)})$,*

*where $\nu_k^{(r)}$ is the non-centrality parameter at time $k$ that depends only on the true parameters, and $M^{(r)} = |\mathcal{K}_r|$ is the difference in the cardinalities of the full and reduced support sets.*

The proof of Corollary 2.1 is presented in Section E.2, with emphasis on the points of departure from the proof Theorem 2.

## E.3 General Case: Testing Nested Hypotheses with History-Dependent Models

The statistical tests proposed in this work have been developed specifically to determine if $r^{\text{th}}$-order coordinated spiking occurs by chance in ensemble activity. However, our proposed methods may be adjusted to test other nested hypotheses about ensemble activity, i.e. tests of the form:

$$H_0 : \boldsymbol{\omega}_k = \hat{\boldsymbol{\omega}}_k^{(R)}, \qquad H_1 : \boldsymbol{\omega}_k \neq \hat{\boldsymbol{\omega}}_k^{(R)}, \tag{34}$$

where $\hat{\boldsymbol{\omega}}_k^{(R)}$ is estimated while restricting an arbitrary subset of size $M$ to fixed values. For example, it might be of interest to inspect $r^{\text{th}}$-order interactions amongst subsets of the neuronal assembly – such as all events involving a particular neuron – rather than amongst the entire assembly. Alternatively, one could in principle characterize the significance of simultaneous spiking events individually rather than as groups.

Corollary 2.2, below, states the limiting distributions of the adaptive de-biased difference for arbitrary nested hypotheses which follow almost immediately from Theorem 2, as expounded in Section F.5.

**Corollary 2.2.** *Let $\hat{\boldsymbol{\omega}}_k^{(F)}$ and $\hat{\boldsymbol{\omega}}_k^{(R)}$ respectively be the full and reduced greedily-estimated parameters of the history-dependent model at window $k$, where an arbitrary subset of the reduced parameters are restricted. Then, as $\beta \to 1$,*

*i) under the null hypothesis, $D_{k,\beta}(\hat{\boldsymbol{\omega}}_k^{(F)}, \hat{\boldsymbol{\omega}}_k^{(R)}) \xrightarrow{d} \chi^2(M)$, and*

*ii) under the alternative hypothesis, assuming the difference between the restricted parameters and their full model estimates scales at least as* $\mathcal{O}\left(\sqrt{\frac{1-\beta}{1+\beta}}\right)$*, then* $D_{k,\beta}\left(\hat{\boldsymbol{\omega}}_k^{(F)}, \hat{\boldsymbol{\omega}}_k^{(R)}\right) \xrightarrow{d} \chi^2(M, \nu_k)$,

*where* $\nu_k$ *is the non-centrality parameter at window* $k$ *and the degrees of freedom* $M$ *is the number of the restricted parameters.*

An important consequence of Corollary 2.2 is its connection to adaptive Granger causality analysis for point process models of neuronal spiking. The discretized point process GLM utilized in [23] is a special case of the history-dependent MkPP mGLM in which $C^* = 1$. Hence, Corollary 2.2 establishes that sparse Granger-causal networks can be adaptively inferred using greedy estimation as an alternative to $\ell_1$-regularized estimation, and thus more tractably applied to larger neuronal assemblies.

# F Proofs

This section contains proofs of the theoretical results presented in Sections D–E. Preliminary results – namely, the limiting behavior of the Hessian matrix, $\nabla^2 \ell_k^\beta(\boldsymbol{\omega}_k^0)$, and gradient of the exponentially-weighted total data log-likelihood, $\nabla \ell_k^\beta(\boldsymbol{\omega}_k^0)$ – are established in Section F.1. The proof of Theorem 1 is provided in Section F.2, followed by the proof of Theorem 2 in Section F.3. Proofs of the corollaries to Theorem 2 are presented next; Corollary 2.1 is addressed in Section F.4 and Corollary 2.2 in Section F.5.

## F.1 Preliminary Results

The limiting behaviors of $\nabla^2 \ell_k^\beta(\boldsymbol{\omega}_k^0)$ and $\nabla \ell_k^\beta(\boldsymbol{\omega}_k^0)$ are characterized in Propositions 1 and 2, respectively. Limits are evaluated over a monotonically increasing sequence $\{\beta_l\}_{l=1}^\infty$ that converges to 1; for notational convenience, the shorthand $\beta \to 1$ has been used throughout. For simplicity and without loss of generality, we analyze the case that the windows over which the model is piece-wise constant are of size $W = 1$ and indexed $i = k - N + 1, \ldots, k$.

**Proposition 1.** *As* $\beta \to 1$,

$$\nabla^2 \ell_k^\beta(\boldsymbol{\omega}_k^0) \to \mathbb{E}\left[\nabla^2 \ell_i(\boldsymbol{\omega}_k)\right] =: -\boldsymbol{\mathcal{I}}_k$$

*Proof.* First, note the following decomposition of the Hessian:

$$\nabla^2 \ell_k^\beta(\boldsymbol{\omega}_k^0) = (1-\beta) \sum_{i=k-N+1}^{k} \beta^{k-i} \nabla^2 \ell_i(\boldsymbol{\omega}_k^0).$$

Taking $\boldsymbol{V}_i = \beta^{k-i} \nabla^2 \ell_i(\boldsymbol{\omega}_k^0)$ and the sequence $c_\beta := \frac{1}{1-\beta}$ that tends to $\infty$ as $\beta \to 1$, a version of the LLN for $\phi$-mixing random variables, [24], is invoked to find that

$$\begin{aligned}
\nabla^2 \ell_k^\beta(\boldsymbol{\omega}_k^0) - \mathbb{E}\left[\nabla^2 \ell_k^\beta(\boldsymbol{\omega}_k^0)\right] &= (1-\beta) \sum_{i=k-N+1}^{k} \left(\beta^{k-i} \nabla^2 \ell_i(\boldsymbol{\omega}_k^0) - \beta^{k-i} \mathbb{E}\left[\nabla^2 \ell_i(\boldsymbol{\omega}_k^0)\right]\right) \\
&= c_\beta^{-1} \sum_{i=k-N+1}^{k} \left(\boldsymbol{V}_i - \mathbb{E}\left[\boldsymbol{V}_i\right]\right) \xrightarrow{a.s.} 0.
\end{aligned}$$

The limiting behavior of the Hessian is thus given by

$$\begin{aligned}
\mathbb{E}\left[\nabla^2 \ell_k^\beta(\boldsymbol{\omega}_k^0)\right] &= \lim_{\beta \to 1} (1-\beta) \sum_{i=1}^{k} \beta^{k-i} \mathbb{E}\left[\nabla^2 \ell_i(\boldsymbol{\omega}_k^0)\right] \\
&= \mathbb{E}\left[\nabla^2 \ell_i(\boldsymbol{\omega}_k^0)\right].
\end{aligned}$$

From the Fisher information equality, we have that $\mathbb{E}\left[\nabla^2 \ell_i(\boldsymbol{\omega}_k^0)\right] = -\boldsymbol{\mathcal{I}}_k$ □

Next, we establish the asymptotic normality of the score function.

**Proposition 2.** *As* $\beta \to 1$,

$$\sqrt{\frac{1+\beta}{1-\beta}} \nabla \ell_k^\beta(\boldsymbol{\omega}_k^0) \to \mathcal{N}\left(\mathbf{0}, \boldsymbol{\mathcal{I}}_k\right)$$

*Proof.* The result is established by invoking a version of the CLT for martingales [24, 25].

First, recall that the gradient with respect to the parameters of the $m^{\text{th}}$ mark,

$$\begin{aligned}\frac{1}{1-\beta}\nabla_{\boldsymbol{\omega}^{(m)}}\ell_k^{\beta}(\boldsymbol{\omega}_k^0) &= \sum_{i=k-N+1}^{k} \beta^{k-i}\nabla_{\boldsymbol{\omega}^{(m)}}\ell_i(\boldsymbol{\omega}_k^0) \\ &= \sum_{i=k-N+1}^{k} \beta^{k-i}\boldsymbol{X}_i{}^{\top}\left[\boldsymbol{n}_i^{*(m)} - \boldsymbol{\lambda}_i^{*(m)}(\boldsymbol{\omega}_k^0)\Delta\right],\end{aligned} \tag{35}$$

and that the covariates $\boldsymbol{X}_t$ are the ensemble spiking history $\{n_j^{(c)}\}_{c=1,j=t-p}^{C,t-1}$. Let $\mathcal{F}_t$ be the $\sigma$-field generated by $\{n_t^{(c)}\}_{c=1,t=t-p}^{C,t-1}$. It is straightforward to verify that each element of $\nabla\ell_k^{\beta}(\boldsymbol{\omega}_k^0)$ is the sum of martingale differences with respect to the filtration $\{\mathcal{F}_t\}_{t=1}^{k}$.

Employing the Cramer-Wold device, it will suffice to characterize the limiting distribution of $Z_k^{\beta} = \boldsymbol{z}^{\top}\left(\frac{1}{1-\beta}\nabla\ell_k^{\beta}(\boldsymbol{\omega}_k^0)\right)$ for an arbitrary unit vector $\boldsymbol{z}$. Let

$$\boldsymbol{U}_t := \beta^{k-t}\nabla\ell_t(\boldsymbol{\omega}_k^0), \quad \boldsymbol{V}_t := \beta^{2(k-t)}\nabla^2\ell_t(\boldsymbol{\omega}_k^0), \tag{36}$$

so that we may write

$$\frac{1}{1-\beta}\nabla\ell_k^{\beta}(\boldsymbol{\omega}_k^0) = \sum_{t=k-N+1}^{k}\boldsymbol{U}_t. \tag{37}$$

Defining $Y_t = Z_t^{\beta} - Z_{t-1}^{\beta} = \boldsymbol{z}^{\top}\boldsymbol{U}_t$, note that $\mathbb{E}\left[Y_t|\,\mathcal{F}_{t-1}\right] = \boldsymbol{z}^{\top}\mathbb{E}\left[\boldsymbol{U}_t|\,\mathcal{F}_{t-1}\right] = 0$; thus, $Z_k^{\beta}$ is a martingale. Also, noting that

$$\mathbb{E}\left[Y_t^2|\mathcal{F}_{t-1}\right] = \boldsymbol{z}^{\top}\mathbb{E}\left[\boldsymbol{U}_t\boldsymbol{U}_t{}^{\top}|\mathcal{F}_{t-1}\right]\boldsymbol{z} = \boldsymbol{z}^{\top}\mathbb{E}\left[-\boldsymbol{V}_t|\mathcal{F}_{t-1}\right]\boldsymbol{z}, \tag{38}$$

we define

$$\begin{aligned} s_{\beta}^2 := \mathbb{E}\left[{Z_k^{\beta}}^2\right] &= \sum_t \mathbb{E}\left[{Y_t}^2\right] \\ &= \boldsymbol{z}^{\top}\mathbb{E}\left[-\sum_t \boldsymbol{V}_t\right]\boldsymbol{z} \\ &= \frac{1}{1-\beta^2}\boldsymbol{z}^{\top}\boldsymbol{\mathcal{I}}_k\boldsymbol{z}.\end{aligned} \tag{39}$$

Thus, we must establish that $Z_k^{\beta}/s_{\beta} \xrightarrow{d} \mathcal{N}(0,1)$ as $\beta \to 1$. The version of CLT for martingales in [25] stipulates this result if the following two conditions hold:

(i) ${s_{\beta}^{-2}}\sum_t \mathbb{E}\left[{Y_t}^2|\mathcal{F}_{t-1}\right] \xrightarrow{p} 1$, and

(ii) ${s_{\beta}^{-2}}\sum_t \mathbb{E}\left[{Y_t}^2\mathbb{1}\left\{|Y_t| \geq \varepsilon s_{\beta}\right\}\right] \to 0, \forall\varepsilon > 0$,

where $\mathbb{1}\{\cdot\}$ denotes the indicator function.

We first address condition (i). Substituting for $s_{\beta}$ and ${Y_t}^2$, the condition is rewritten as

$$\frac{(1-\beta^2)\sum_t \boldsymbol{z}^{\top}\mathbb{E}\left[-\boldsymbol{V}_t|\mathcal{F}_{t-1}\right]\boldsymbol{z}}{\boldsymbol{z}^{\top}\boldsymbol{\mathcal{I}}_k\boldsymbol{z}} \to 1. \tag{40}$$

Letting $\rho = \beta^2$, and using the definition of $\boldsymbol{V}_t$, the condition may be equivalently expressed as

$$\frac{(1-\rho)\boldsymbol{z}^{\top}\sum_t \rho^{k-t}\left(\mathbb{E}\left[-\nabla^2\ell_t(\boldsymbol{\omega}_k^0)|\mathcal{F}_{t-1}\right] - \mathbb{E}\left[-\nabla^2\ell_k(\boldsymbol{\omega}_k^0)\right]\right)\boldsymbol{z}}{\boldsymbol{z}^{\top}\mathbb{E}\left[-\nabla^2\ell_k(\boldsymbol{\omega}_k^0)\right]\boldsymbol{z}} \xrightarrow{p} 0, \tag{41}$$

where we have used the Fisher information equality; this condition is implied by monotone convergence and the result of Proposition 1.

We next consider condition (ii), also known as the Lindeberg condition. As $\beta \to 1$, note that

$$s_\beta = \left(\frac{1}{1-\beta^2}\boldsymbol{z}^\top \boldsymbol{\mathcal{I}}_k \boldsymbol{z}\right)^{\frac{1}{2}} \to \infty,$$

while

$$|Y_t| = \left|\boldsymbol{z}^\top \beta^{k-t} \nabla \ell_t(\boldsymbol{\omega}_k^0)\right| \to \left|\boldsymbol{z}^\top \nabla \ell_t(\boldsymbol{\omega}_k^0)\right|.$$

Thus, $\mathbb{1}\{|Y_t| \geq \varepsilon s_\beta\} \to 0$ for all $\varepsilon > 0$, and the Lindeberg condition holds, thereby proving $Z_k^\beta / s_\beta \xrightarrow{d} \mathcal{N}(0,1)$. To relate the statement of the proposition to $Z_k^\beta / s_\beta$ more explicitly, observe that

$$\begin{aligned} \frac{Z_k^\beta}{s_\beta} &= \frac{\boldsymbol{z}^\top \nabla \ell_k^\beta(\boldsymbol{\omega}_k^0)/(1-\beta)}{\left(\boldsymbol{z}^\top \boldsymbol{\mathcal{I}}_k \boldsymbol{z}/(1-\beta^2)\right)^{1/2}} \\ &= \sqrt{\frac{1+\beta}{1-\beta}} \frac{\boldsymbol{z}^\top \nabla \ell_k^\beta(\boldsymbol{\omega}_k^0)}{\sqrt{\boldsymbol{z}^\top \boldsymbol{\mathcal{I}}_k \boldsymbol{z}}}. \end{aligned} \tag{42}$$

□

## F.2 Proof of Theorem 1

*Proof.* To begin, we analyze the de-sparsified estimates $\hat{\boldsymbol{w}}_k = \hat{\boldsymbol{\omega}}_k - \hat{\Theta}_k \nabla \ell_k^\beta(\hat{\boldsymbol{\omega}}_k)$ element-wise, attending particularly to the latter term of the right hand side. For the $j^{\text{th}}$ element,

$$\begin{aligned} (\hat{\Theta}_k)_j \nabla \ell_k^\beta(\hat{\boldsymbol{\omega}}_k) =& (\hat{\Theta}_k)_j \nabla \ell_k^\beta(\boldsymbol{\omega}_k^0) \\ &+ (\hat{\Theta}_k)_j \nabla^2 \ell_k^\beta(\hat{\boldsymbol{\omega}}_k)\left(\hat{\boldsymbol{\omega}}_k - \boldsymbol{\omega}_k^0\right) + \Delta_1^{(j)}, \end{aligned} \tag{43}$$

where

$$\Delta_1^{(j)} = (\hat{\Theta}_k)_j \left(\nabla^2 \ell_k^\beta(\boldsymbol{\omega}_k^0) - \nabla^2 \ell_k^\beta(\hat{\boldsymbol{\omega}}_k)\right)\left(\hat{\boldsymbol{\omega}}_k - \boldsymbol{\omega}_k^0\right). \tag{44}$$

Note that based on the expansion in Eq. (43), the difference between the desparsified and maximimum likelihood estimates (for the $j^{\text{th}}$ component)

$$\begin{aligned} (\hat{\boldsymbol{w}}_k)_j - (\boldsymbol{\omega}_k^0)_j &= (\hat{\boldsymbol{\omega}}_k)_j - (\boldsymbol{\omega}_k^0)_j - (\hat{\Theta}_k)_j \nabla \ell_k^\beta(\hat{\boldsymbol{\omega}}_k) \\ &= (\hat{\boldsymbol{\omega}}_k)_j - (\boldsymbol{\omega}_k^0)_j - (\hat{\Theta}_k)_j \nabla \ell_k^\beta(\boldsymbol{\omega}_k^0) \\ &\quad - (\hat{\Theta}_k)_j \nabla^2 \ell_k^\beta(\hat{\boldsymbol{\omega}}_k)(\hat{\boldsymbol{\omega}}_k - \boldsymbol{\omega}_k^0) - \Delta_1^{(j)} \\ &= -(\hat{\Theta}_k)_j \nabla \ell_k^\beta(\boldsymbol{\omega}_k^0) - \Delta_2^{(j)}, \end{aligned} \tag{45}$$

where

$$\Delta_2^{(j)} = \Delta_1^{(j)} + \left((\hat{\Theta}_k)_j \nabla^2 \ell_k^\beta(\hat{\boldsymbol{\omega}}_k) - \boldsymbol{e}_j^\top\right)(\hat{\boldsymbol{\omega}}_k - \boldsymbol{\omega}_k^0), \tag{46}$$

with $\boldsymbol{e}_j$ a standard basis vector. In the following, we derive uniform upper bounds on both $|\Delta_1^{(j)}|$ and $|\Delta_2^{(j)}|$ for all $j = 1, \ldots, d$.

We reintroduce two notations from conditions (C1) and (C5) to proceed. Recall that the collection of covariate matrices over all windows is denoted as $\boldsymbol{X} = \left[\boldsymbol{X}_1{}^\top, \ldots, \boldsymbol{X}_k{}^\top\right]^\top$; letting $\tilde{\boldsymbol{X}}_i$ denote the matrix $\boldsymbol{X}_i$ tiled horizontally $C^*$ times, we also define the $T \times d$ matrix $\tilde{\boldsymbol{X}} = \left[\tilde{\boldsymbol{X}}_1^\top, \ldots, \tilde{\boldsymbol{X}}_k^\top\right]^\top$. Thus, for $i = 1, \ldots, k$, we defined $\boldsymbol{z}_i := \tilde{\boldsymbol{X}}_i \boldsymbol{\omega}_k$, and the log-likelihood function for the $i^{\text{th}}$ window can be written as $\ell_i(\boldsymbol{\omega}_k) = \ell_i(\boldsymbol{z}_i)$.

For the $i^{\mathsf{th}}$ window, note that

$$\nabla_{\boldsymbol{z}}\ell_i(\hat{\boldsymbol{z}}_i) = \nabla_{\boldsymbol{z}}\ell_i(\boldsymbol{z}_i^0) + \nabla^2_{\boldsymbol{z}}\ell_i(\tilde{\boldsymbol{z}}_i)\left(\hat{\boldsymbol{z}}_i - \boldsymbol{z}_i^0\right), \tag{47}$$

for some $\tilde{\boldsymbol{z}}_i$ such that $\|\tilde{\boldsymbol{z}}_i - \hat{\boldsymbol{z}}_i\|_2 \leq \left\|\hat{\boldsymbol{z}}_i - \boldsymbol{z}_i^0\right\|_2$. Using condition (C1), we have that

$$\begin{aligned}\left\|\left(\nabla^2_{\boldsymbol{z}}\ell_i(\tilde{\boldsymbol{z}}_i) - \nabla^2_{\boldsymbol{z}}\ell_i(\hat{\boldsymbol{z}}_i)\right)\left(\hat{\boldsymbol{z}}_i - \boldsymbol{z}_i^0\right)\right\|_2 &\leq \|\tilde{\boldsymbol{z}}_i - \hat{\boldsymbol{z}}_i\|_2 \left\|\hat{\boldsymbol{z}}_i - \boldsymbol{z}_i^0\right\|_2 \\ &\leq \left\|\hat{\boldsymbol{z}}_i - \boldsymbol{z}_i^0\right\|_2^2.\end{aligned} \tag{48}$$

Additionally, note that, by the chain rule for derivatives,

$$\nabla^2_{\boldsymbol{\omega}}\ell_i(\boldsymbol{\omega}_k) = \tilde{\boldsymbol{X}}_i^\top \nabla^2_{\boldsymbol{z}}\ell_i(\boldsymbol{z}_i)\tilde{\boldsymbol{X}}_i. \tag{49}$$

Using these two relations, a uniform upper bound for $\{\Delta_1^{(j)}\}_{j=1}^d$ is derived as follows. The residual is expanded as

$$\Delta_1^{(j)} = (1-\beta)\sum_{i=1}^{k}\beta^{k-i}(\hat{\Theta}_k)_j\tilde{\boldsymbol{X}}_i^\top\left(\nabla^2_{\boldsymbol{z}}\ell_i(\boldsymbol{z}_i^0) - \nabla^2_{\boldsymbol{z}}\ell_i(\hat{\boldsymbol{z}}_i)\right)\tilde{\boldsymbol{X}}_i(\hat{\boldsymbol{\omega}}_k - \boldsymbol{\omega}_k^0). \tag{50}$$

First the $i^{\mathsf{th}}$ term in the summation, neglecting $\beta^{k-i}$, is addressed:

$$\begin{aligned}&\left|(\hat{\Theta}_k)_j\tilde{\boldsymbol{X}}_i^\top\left(\nabla^2_{\boldsymbol{z}}\ell_i(\boldsymbol{z}_i^0) - \nabla^2_{\boldsymbol{z}}\ell_i(\hat{\boldsymbol{z}}_i)\right)\tilde{\boldsymbol{X}}_i(\hat{\boldsymbol{\omega}}_k - \boldsymbol{\omega}_k^0)\right| \\ &\leq \left\|(\hat{\Theta}_k)_j\tilde{\boldsymbol{X}}_i^\top\right\|_\infty \left\|\left(\nabla^2_{\boldsymbol{z}}\ell_i(\boldsymbol{z}_i^0) - \nabla^2_{\boldsymbol{z}}\ell_i(\hat{\boldsymbol{z}}_i)\right)\tilde{\boldsymbol{X}}_i(\hat{\boldsymbol{\omega}}_k - \boldsymbol{\omega}_k^0)\right\|_1 \\ &\leq \left\|(\hat{\Theta}_k)_j\tilde{\boldsymbol{X}}_i^\top\right\|_\infty \sqrt{d}\left\|\left(\nabla^2_{\boldsymbol{z}}\ell_i(\boldsymbol{z}_i^0) - \nabla^2_{\boldsymbol{z}}\ell_i(\hat{\boldsymbol{z}}_i)\right)\tilde{\boldsymbol{X}}_i(\hat{\boldsymbol{\omega}}_k - \boldsymbol{\omega}_k^0)\right\|_2 \\ &\leq \left\|\hat{\Theta}_k\tilde{\boldsymbol{X}}^\top\right\|_\infty \sqrt{d}\left\|\tilde{\boldsymbol{X}}_i(\hat{\boldsymbol{\omega}}_k - \boldsymbol{\omega}_k^0)\right\|_2^2,\end{aligned}$$

where the first inequality is a consequence of Hölder's inequality, the second is due to the equivalence of norms, and the third follows by invoking condition (C1), as in Eq. (48). Using the fact that $\beta < 1$, it follows that

$$\begin{aligned}\left|\Delta_1^{(j)}\right| &\leq (1-\beta)\sum_{i=1}^{k}\beta^{k-i}\left\|\hat{\Theta}_k\tilde{\boldsymbol{X}}^\top\right\|_\infty \sqrt{d}\left\|\tilde{\boldsymbol{X}}_i(\hat{\boldsymbol{\omega}}_k - \boldsymbol{\omega}_k^0)\right\|_2^2 \\ &\leq \left\|\hat{\Theta}_k\tilde{\boldsymbol{X}}^\top\right\|_\infty \sqrt{d}\left\|\tilde{\boldsymbol{X}}(\hat{\boldsymbol{\omega}}_k - \boldsymbol{\omega}_k^0)\right\|_2^2.\end{aligned} \tag{51}$$

Consequently, the term $|\Delta_2^{(j)}|$ is upper-bounded as follows:

$$\begin{aligned}\left|\Delta_2^{(j)}\right| \leq &\left\|\hat{\Theta}_k\tilde{\boldsymbol{X}}^\top\right\|_\infty \sqrt{d}\left\|\tilde{\boldsymbol{X}}(\hat{\boldsymbol{\omega}}_k - \boldsymbol{\omega}_k^0)\right\|_2^2 \\ &+ \left\|(\hat{\Theta}_k)_j\nabla^2\ell_k^\beta(\hat{\boldsymbol{\omega}}_k) - \boldsymbol{e}_j^\top\right\|_\infty \left\|\hat{\boldsymbol{\omega}}_k - \boldsymbol{\omega}_k^0\right\|_1.\end{aligned} \tag{52}$$

Invoking conditions (C5) and (C6),

$$\begin{aligned}\left\|\hat{\Theta}_k\tilde{\boldsymbol{X}}^\top\right\|_\infty \sqrt{d}\left\|\tilde{\boldsymbol{X}}(\hat{\boldsymbol{\omega}}_k - \boldsymbol{\omega}_k^0)\right\|_2^2 &= \mathcal{O}_{\mathbb{P}}\left(c_1\sqrt{d}(1-\beta)\log(s)\log(d)\right) \\ &= o_{\mathbb{P}}(1),\end{aligned} \tag{53}$$

and hence, $\left|\Delta_1^{(j)}\right|$ is negligible for each $j = 1, \ldots, d$. Similarly, condition (C2) implies that $\left\|\hat{\boldsymbol{\omega}}_k - \boldsymbol{\omega}_k^0\right\|_1 = \mathcal{O}_{\mathbb{P}}\left(\sqrt{1-\beta}\sqrt{\log(s)\log(d)}\sqrt{d}\right) = o_{\mathbb{P}}(1)$; invoking the consistency of

the estimator $\hat{\Theta}_k$, the second part of condition (C3), it follows that $\left|\Delta_2^{(j)}\right| = o_{\mathbb{P}}(1)$. Of course, this conclusion follows immediately from Eq. (52) if $\nabla^2 \ell_k^\beta$ is invertible, as per the first part of condition (C3). Thus, we have shown that
$\hat{\boldsymbol{w}}_k - \boldsymbol{\omega}_k^0 = -(\hat{\Theta}_k)\nabla \ell_k^\beta(\boldsymbol{\omega}_k^0) + o_{\mathbb{P}}(1) \cdot \mathbf{1}$.

Proposition 1 establishes that $\nabla^2 \ell_k^\beta(\boldsymbol{\omega}_k^0) \to \mathbb{E}[\nabla^2 \ell_i(\boldsymbol{\omega}_k)]$, or equivalently that $\hat{\Sigma}_k \to \Sigma_k$; invoking condition (C3), it follows that $\hat{\Theta}_k \to \Sigma_k^{-1}$. Recalling that this is a sequential limit in $\beta$, if $\hat{\Sigma}_k$ is invertible for each $\beta$, $\hat{\Theta}_k \to \Sigma_k^{-1}$ by the continuous mapping theorem. Alternatively, if $\hat{\Theta}_k$ is not invertible, the statement follows directly by second part of condition (C3). Proposition 2 establishes the asymptotic normality of $\nabla \ell_k^\beta(\boldsymbol{\omega}_k^0)$; in conjunction with the aforementioned limiting behavior of $\hat{\Theta}_k$ and Slutsky's theorem, we thus conclude that $\sqrt{\frac{1+\beta}{1-\beta}}\left(\hat{\boldsymbol{w}}_k - \boldsymbol{\omega}_k^0\right)$ is asymptotically normal with zero-mean and covariance matrix $\Sigma_k^{-1}\boldsymbol{\mathcal{I}}_k\Sigma_k^{-1} = \boldsymbol{\mathcal{I}}_k^{-1}$. □

## F.3 Proof of Theorem 2

*Proof.* We begin with the adaptive de-biased deviance statistic for the estimated parameters $\hat{\boldsymbol{\omega}}_k$ and the true parameters $\boldsymbol{\omega}_k$:

$$D_{k,\beta}(\hat{\boldsymbol{\omega}}_k, \boldsymbol{\omega}_k) = \left(\frac{1+\beta}{1-\beta}\right)\left[2\left(\ell_k^\beta(\hat{\boldsymbol{\omega}}_k) - \ell_k^\beta(\boldsymbol{\omega}_k)\right) - \mathscr{B}_k\right], \tag{54}$$

where $\mathscr{B}_k := \nabla \ell_k^\beta(\hat{\boldsymbol{\omega}}_k)^\top \left(\nabla^2 \ell_k^\beta(\boldsymbol{\omega}_k)\right)^{-1} \nabla \ell_k^\beta(\hat{\boldsymbol{\omega}}_k)$. To write the deviance in a more convenient form, we first note the quadratic expansion of $\ell_k^\beta(\boldsymbol{\omega}_k)$:

$$\begin{aligned}\ell_k^\beta(\boldsymbol{\omega}_k) =&\ell_k^\beta(\hat{\boldsymbol{\omega}}_k) + (\boldsymbol{\omega}_k - \hat{\boldsymbol{\omega}}_k)^\top \nabla \ell_k^\beta(\hat{\boldsymbol{\omega}}_k)\\ &+ \frac{1}{2}(\boldsymbol{\omega}_k - \hat{\boldsymbol{\omega}}_k)^\top \left(\nabla^2 \ell_k^\beta(\tilde{\boldsymbol{\omega}}_k)\right)(\boldsymbol{\omega}_k - \hat{\boldsymbol{\omega}}_k).\end{aligned} \tag{55}$$

Substituting into Eq. (54) and expressing the bias correction term $\mathscr{B}_k$ explicitly, the deviance is equivalent to

$$\begin{aligned}\left(\frac{1-\beta}{1+\beta}\right) D_{k,\beta}(\hat{\boldsymbol{\omega}}_k, \boldsymbol{\omega}_k) =&2(\hat{\boldsymbol{\omega}}_k - \boldsymbol{\omega}_k)^\top \nabla \ell_k^\beta(\hat{\boldsymbol{\omega}}_k)\\ &- (\hat{\boldsymbol{\omega}}_k - \boldsymbol{\omega}_k)^\top \left(\nabla^2 \ell_k^\beta(\tilde{\boldsymbol{\omega}}_k)\right)(\hat{\boldsymbol{\omega}}_k - \boldsymbol{\omega}_k)\\ &- \nabla \ell_k^\beta(\hat{\boldsymbol{\omega}}_k)^\top \left(\nabla^2 \ell_k^\beta(\boldsymbol{\omega}_k)\right)^{-1} \nabla \ell_k^\beta(\hat{\boldsymbol{\omega}}_k),\end{aligned} \tag{56}$$

where $\tilde{\boldsymbol{\omega}}_k$ intermediates $\hat{\boldsymbol{\omega}}_k$ and $\boldsymbol{\omega}_k$. By rearranging terms and recalling that the de-sparsified estimate $\hat{\boldsymbol{w}}_k = \hat{\boldsymbol{\omega}}_k - \left(\nabla^2 \ell_k^\beta(\hat{\boldsymbol{\omega}}_k)\right)^{-1} \nabla \ell_k^\beta(\hat{\boldsymbol{\omega}}_k)$, the deviance can be compactly expressed as:

$$\left(\frac{1-\beta}{1+\beta}\right) D_{k,\beta}(\hat{\boldsymbol{\omega}}_k, \boldsymbol{\omega}_k) = -\left(\hat{\boldsymbol{w}}_k - \boldsymbol{\omega}_k\right)^\top \nabla^2 \ell_k^\beta(\boldsymbol{\omega}_k)\left(\hat{\boldsymbol{w}}_k - \boldsymbol{\omega}_k\right) + \Delta_1, \tag{57}$$

with $\Delta_1 = (\hat{\boldsymbol{\omega}}_k - \boldsymbol{\omega}_k)^\top \left(\nabla^2 \ell_k^\beta(\tilde{\boldsymbol{\omega}}_k) - \nabla^2 \ell_k^\beta(\boldsymbol{\omega}_k)\right)(\hat{\boldsymbol{\omega}}_k - \boldsymbol{\omega}_k)$. It can be shown based on the conditions of Theorem 1 that $|\Delta_1| = \mathcal{O}_{\mathbb{P}}\left(\|\hat{\boldsymbol{\omega}}_k - \boldsymbol{\omega}_k\|^3\right) = o_{\mathbb{P}}\left((1-\beta)^{3/2}\right)$, and thus negligible. Once again, negligible terms are summarized as $o_{\mathbb{P}}(1)$ terms. Similar arguments were presented in the course of proving Theorem 1.

We now explicitly define the null and sequence of local alternative hypotheses in order to adapt Davidson and Lever's treatment [22]. Recall first that limits in $\beta$ are

understood to be sequential limits; i.e., they are evaluated for the sequence $\{\beta_l\}_{l=1}^{\infty}$ where $\beta_l \to 1$ as $l \to \infty$. Then, for window $k$, we test the null hypothesis $H_{0,k} : \boldsymbol{\omega}_k^0 = (\boldsymbol{\omega}_{0,k}, \boldsymbol{\omega}_{1,k})$ against the sequence of local alternative hypotheses $\left\{H_{1,k}^{\beta_l} : \boldsymbol{\omega}_k^{\beta_l} = \left(\boldsymbol{\omega}_{0,k}^*, \boldsymbol{\omega}_{1,k}^{\beta_l}\right)\right\}_{l=1}^{\infty}$, where $\boldsymbol{\omega}_{1,k}^{\beta_l} = \boldsymbol{\omega}_{1,k} + \sqrt{\frac{1-\beta_l}{1+\beta_l}}\boldsymbol{\delta}_k$; the limiting true parameter vector under the alternative is denoted by $\boldsymbol{\omega}_k^*$. The partition of the $d-$dimensional parameter vector corresponds to the free parameters under the null hypothesis $(\boldsymbol{\omega}_{0,k})$ and the restricted sub-vector $(\boldsymbol{\omega}_{1,k})$. Thus, the statistical test seeks to detect local perturbations of order $\mathcal{O}\left(\sqrt{\frac{1-\beta}{1+\beta}}\right)$ from the null hypothesis. For the statistical inference of $r^{\text{th}}$-order coordination, $\boldsymbol{\omega}_{1,k}$ corresponds to the base rate parameters of $r^{\text{th}}$-order events, the number of which is denoted by $M^{(r)}$; however, since a similar partition can be made for any nested hypothesis test, the result shown here generalizes.

We now establish the limiting behavior of the de-biased deviance difference under the null hypothesis. By Proposition 1, $\nabla^2 \ell_k^{\beta}(\boldsymbol{\omega}_k) \to -\mathcal{I}_k$. Additionally, by Proposition 2, $\sqrt{\frac{1+\beta}{1-\beta}}\nabla \ell_k^{\beta}(\hat{\boldsymbol{\omega}}_k) \to \mathcal{N}(\mathbf{0}, \mathcal{I}_k)$. Thus, recalling the definition of the the de-sparsified estimate $\hat{\boldsymbol{w}}_k$ and invoking Slutsky's Theorem, $\sqrt{\frac{1+\beta}{1-\beta}}(\hat{\boldsymbol{w}}_k - \boldsymbol{\omega}_k) \to \mathcal{N}(\mathbf{0}, \mathcal{I}_k^{-1})$ and consequently, the deviance $[D_{k,\beta}(\hat{\boldsymbol{\omega}}_k, \boldsymbol{\omega}_k)|H_{0,k}] \to \chi^2(d)$, asymptotically following a $\chi^2$ distribution with $d$ degrees of freedom. Finally, invoking classical results for likelihood ratio tests of nested models [26, 27], we conclude that under the null hypothesis, the adaptive de-biased deviance difference converges in distribution to a $\chi^2$ random variable with $M^{(r)}$ degrees of freedom:

$$\left[D_{k,\beta}^{(r)}\left(\hat{\boldsymbol{\omega}}_k^{(F)}, \hat{\boldsymbol{\omega}}_k^{(R)}\right)\middle|H_{0,k}\right] \to \chi^2(M^{(r)}) \tag{58}$$

It remains to establish the limiting behavior of the adaptive de-biased deviance difference under the sequence of alternative hypotheses. Though Proposition 1 establishes that $\nabla^2 \ell_k^{\beta}(\boldsymbol{\omega}_k^*) \to -\boldsymbol{\mathcal{I}}_k^*$, the asymptotic normality of $\sqrt{\frac{1+\beta}{1-\beta}}(\hat{\boldsymbol{w}}_k - \boldsymbol{\omega}_k^*)$ does not follow as readily. To establish asymptotic normality, we first derive an alternative expression for the difference $(\hat{\boldsymbol{w}}_k - \boldsymbol{\omega}_k^*)$ using the following two Taylor expansions. The gradient of the log-likelihood evaluated at $\hat{\boldsymbol{\omega}}_k$ and $\boldsymbol{\omega}_k^{\beta}$ may equivalently be written as:

$$\begin{aligned} \nabla \ell_k^{\beta}(\hat{\boldsymbol{\omega}}_k) &= \nabla \ell_k^{\beta}(\boldsymbol{\omega}_k^*) + \nabla^2 \ell_k^{\beta}(\boldsymbol{\omega}_k^*)\left(\hat{\boldsymbol{\omega}}_k - \boldsymbol{\omega}_k^*\right) + \Delta_2 \\ \nabla \ell_k^{\beta}(\boldsymbol{\omega}_k^{\beta}) &= \nabla \ell_k^{\beta}(\boldsymbol{\omega}_k^*) + \nabla^2 \ell_k^{\beta}(\boldsymbol{\omega}_k^*)\left(\boldsymbol{\omega}_k^{\beta} - \boldsymbol{\omega}_k^*\right) + \Delta_3, \end{aligned} \tag{59}$$

respectively. The remainder terms

$$\Delta_2 := \left(\nabla^2 \ell_k^{\beta}(\boldsymbol{\omega}_k') - \nabla^2 \ell_k^{\beta}(\boldsymbol{\omega}_k^*)\right)\left(\hat{\boldsymbol{\omega}}_k - \boldsymbol{\omega}_k^*\right)$$

and

$$\Delta_3 := \left(\nabla^2 \ell_k^{\beta}(\boldsymbol{\omega}_k'') - \nabla^2 \ell_k^{\beta}(\boldsymbol{\omega}_k^*)\right)\left(\boldsymbol{\omega}_k^{\beta} - \boldsymbol{\omega}_k^*\right),$$

are negligible at rate $\|\hat{\boldsymbol{\omega}}_k - \boldsymbol{\omega}_k^*\|^2 = o_{\mathbb{P}}(1-\beta)$, which can also be shown based on the conditions of Theorem 1. Thus, we can rewrite

$$\begin{aligned} \hat{\boldsymbol{w}}_k - \boldsymbol{\omega}_k^* &= \hat{\boldsymbol{\omega}}_k - \boldsymbol{\omega}_k^* - \left(\nabla^2 \ell_k^{\beta}(\boldsymbol{\omega}_k^*)\right)^{-1} \nabla \ell_k^{\beta}(\hat{\boldsymbol{\omega}}_k) \\ &= -\left(\nabla^2 \ell_k^{\beta}(\boldsymbol{\omega}_k^*)\right)^{-1} \nabla \ell_k^{\beta}(\boldsymbol{\omega}_k^*) + o_{\mathbb{P}}(1) \\ &= \boldsymbol{\omega}_k^{\beta} - \boldsymbol{\omega}_k^* - \left(\nabla^2 \ell_k^{\beta}(\boldsymbol{\omega}_k^*)\right)^{-1} \nabla \ell_k^{\beta}(\boldsymbol{\omega}_k^{\beta}) + o_{\mathbb{P}}(1). \end{aligned} \tag{60}$$

Denoting $\boldsymbol{\omega}_k^\beta - \boldsymbol{\omega}_k^*$ as $\tilde{\boldsymbol{\delta}}_k := \left[\mathbf{0}^\top, \sqrt{\frac{1-\beta}{1+\beta}}\boldsymbol{\delta}_k^\top\right]^\top$, invoking Propositions 1 and 2, by Slutsky's theorem we find that $\sqrt{\frac{1+\beta}{1-\beta}}\left(\hat{\boldsymbol{w}}_k - \boldsymbol{\omega}_k^*\right) \to \mathcal{N}\left(\tilde{\boldsymbol{\delta}}_k, (\boldsymbol{\mathcal{I}}_k^*)^{-1}\right)$. In contrast to the null case, the limiting normal distribution has non-zero mean; thus, we employ Davidson and Lever's approach [22] to establish the limiting behavior of the adaptive de-biased deviance difference.

To proceed, we first decompose the Fisher information matrix in accordance with the partition of the parameter vector introduced earlier. The parameters $\boldsymbol{\omega}_k^*$ were partitioned into $\boldsymbol{\omega}_{0,k}^*$, corresponding to the parameters free under the null hypothesis, and $\boldsymbol{\omega}_{1,k}^*$, the parameters restricted under the null hypothesis; $\boldsymbol{\mathcal{I}}_k^*$ is similarly decomposed as:

$$\boldsymbol{\mathcal{I}}_k^* = \begin{bmatrix} \boldsymbol{\mathcal{I}}_{0,0,k}^* & \boldsymbol{\mathcal{I}}_{0,1,k}^* \\ \boldsymbol{\mathcal{I}}_{1,0,k}^* & \boldsymbol{\mathcal{I}}_{1,1,k}^* \end{bmatrix},$$

where $\boldsymbol{\mathcal{I}}_{0,1,k}^* = {\boldsymbol{\mathcal{I}}_{1,0,k}^*}^\top$. Utilizing the quadratic form of the de-biased deviance in Eq. (57) and invoking Proposition 1, the adaptive de-biased deviance difference is expressed as:

$$\begin{aligned} D_{k,\beta}^{(r)}\left(\hat{\boldsymbol{\omega}}_k^{(F)}, \hat{\boldsymbol{\omega}}_k^{(R)}\right) &= \left(\frac{1+\beta}{1-\beta}\right)\left(\hat{\boldsymbol{w}}_k^{(F)} - \boldsymbol{\omega}_k^*\right)^\top \boldsymbol{\mathcal{I}}_k^* \left(\hat{\boldsymbol{w}}_k^{(F)} - \boldsymbol{\omega}_k^*\right) \\ &- \left(\frac{1+\beta}{1-\beta}\right)\left(\hat{\boldsymbol{w}}_{0,k}^{(R)} - \boldsymbol{\omega}_{0,k}^*\right)^\top \boldsymbol{\mathcal{I}}_{0,0,k}^* \left(\hat{\boldsymbol{w}}_{0,k}^{(R)} - \boldsymbol{\omega}_{0,k}^*\right) \\ &+ o_{\mathbb{P}}(1). \end{aligned} \tag{61}$$

In the following steps, an equivalent expression for the reduced model deviance is derived in terms of $\left(\hat{\boldsymbol{w}}_k^{(F)} - \boldsymbol{\omega}_k^*\right)$. Recalling the Taylor expansion of $\nabla \ell_k^\beta(\hat{\boldsymbol{\omega}}_k)$ around $\boldsymbol{\omega}_k^*$ in Eq. (59), we see that

$$\hat{\boldsymbol{w}}_k - \boldsymbol{\omega}_k^* = -\left(\nabla^2 \ell_k^\beta(\boldsymbol{\omega}_k^*)\right)^{-1} \nabla \ell_k^\beta(\boldsymbol{\omega}_k^*) + o_{\mathbb{P}}(1-\beta),$$

and that

$$\nabla \ell_k^\beta(\boldsymbol{\omega}_k^*) = \boldsymbol{\mathcal{I}}_k^*\left(\hat{\boldsymbol{w}}_k - \boldsymbol{\omega}_k^*\right) + o_{\mathbb{P}}(1-\beta),$$

having invoked Proposition 1. It follows that the partial gradient $\nabla_{\boldsymbol{\omega}_{0,k}} \ell_k^\beta(\boldsymbol{\omega}_k^*) = \boldsymbol{\mathcal{I}}_{0,0,k}^*\left(\hat{\boldsymbol{w}}_{0,k} - \boldsymbol{\omega}_{0,k}^*\right) + o_{\mathbb{P}}(1-\beta)$, and so the second term of Eq. (61) is equivalent to

$$\left(\frac{1+\beta}{1-\beta}\right)\left(\nabla_{\boldsymbol{\omega}_{0,k}} \ell_k^\beta(\boldsymbol{\omega}_k^*)\right)^\top \left(\boldsymbol{\mathcal{I}}_{0,0,k}^*\right)^{-1}\left(\nabla_{\boldsymbol{\omega}_{0,k}} \ell_k^\beta(\boldsymbol{\omega}_k^*)\right) + o_{\mathbb{P}}(1). \tag{62}$$

Note that the partial gradient is a subvector of the gradient, i.e., $\nabla_{\boldsymbol{\omega}_{0,k}} \ell_k^\beta(\boldsymbol{\omega}_k^*) = \left(\nabla \ell_k^\beta(\boldsymbol{\omega}_k^*)\right)_0$. Hence,

$$\begin{aligned} \nabla_{\boldsymbol{\omega}_{0,k}} \ell_k^\beta(\boldsymbol{\omega}_k^*) &= \left(\boldsymbol{\mathcal{I}}_k^*\left(\hat{\boldsymbol{w}}_k^{(F)} - \boldsymbol{\omega}_k^*\right)\right)_0 + o_{\mathbb{P}}(1-\beta) \\ &= \boldsymbol{\mathcal{I}}_{0,\cdot,k}^*\left(\hat{\boldsymbol{w}}_k^{(F)} - \boldsymbol{\omega}_k^*\right) + o_{\mathbb{P}}(1-\beta), \end{aligned} \tag{63}$$

where $\boldsymbol{\mathcal{I}}_{0,\cdot,k}^* = \left[\boldsymbol{\mathcal{I}}_{0,0,k}^*, \boldsymbol{\mathcal{I}}_{0,1,k}^*\right]$. Equation (62) can then be expressed as

$$\left(\frac{1+\beta}{1-\beta}\right)\left(\hat{\boldsymbol{w}}_k^{(F)} - \boldsymbol{\omega}_k^*\right)^\top \boldsymbol{A}\left(\hat{\boldsymbol{w}}_k^{(F)} - \boldsymbol{\omega}_k^*\right) + o_{\mathbb{P}}(1), \tag{64}$$

where

$$\boldsymbol{A} = \begin{bmatrix} \boldsymbol{\mathcal{I}}_{0,0,k}^* & \boldsymbol{\mathcal{I}}_{0,1,k}^* \\ \boldsymbol{\mathcal{I}}_{1,0,k}^* & \boldsymbol{\mathcal{I}}_{1,0,k}^*\left(\boldsymbol{\mathcal{I}}_{0,0,k}^*\right)^{-1}\boldsymbol{\mathcal{I}}_{0,1,k}^* \end{bmatrix}.$$

Thus, the adaptive de-biased deviance difference is equal to

$$D_{k,\beta}^{(r)} = \left(\frac{1+\beta}{1-\beta}\right) \left(\hat{\boldsymbol{w}}_k^{(F)} - \boldsymbol{\omega}_k^*\right)^\top \left(\boldsymbol{\mathcal{I}}_k^* - \boldsymbol{A}\right) \left(\hat{\boldsymbol{w}}_k^{(F)} - \boldsymbol{\omega}_k^*\right) + o_{\mathbb{P}}(1). \tag{65}$$

Note that

$$\boldsymbol{\mathcal{I}}_k^* - \boldsymbol{A} = \begin{bmatrix} \mathbf{0} & \mathbf{0} \\ \mathbf{0} & \boldsymbol{\mathcal{I}}_{1,1,k}^* - \boldsymbol{\mathcal{I}}_{1,0,k}^* \left(\boldsymbol{\mathcal{I}}_{0,0,k}^*\right)^{-1} \boldsymbol{\mathcal{I}}_{0,1,k}^* \end{bmatrix}, \tag{66}$$

and so, defining $\tilde{\boldsymbol{\mathcal{I}}}_k^* := \boldsymbol{\mathcal{I}}_{1,1,k}^* - \boldsymbol{\mathcal{I}}_{1,0,k}^* \left(\boldsymbol{\mathcal{I}}_{0,0,k}^*\right)^{-1} \boldsymbol{\mathcal{I}}_{0,1,k}^* = \left(\boldsymbol{\mathcal{I}}_k^{*-1}\right)_{1,1}^{-1}$, the deviance difference simplifies to

$$D_{k,\beta}^{(r)} = \left(\frac{1+\beta}{1-\beta}\right) \left(\hat{\boldsymbol{w}}_{1,k}^{(F)} - \boldsymbol{\omega}_{1,k}^*\right)^\top \tilde{\boldsymbol{\mathcal{I}}}_k^* \left(\hat{\boldsymbol{w}}_{1,k}^{(F)} - \boldsymbol{\omega}_{1,k}^*\right) + o_{\mathbb{P}}(1). \tag{67}$$

That $\sqrt{\frac{1+\beta}{1-\beta}} \left(\hat{\boldsymbol{w}}_{1,k}^{(F)} - \boldsymbol{\omega}_{1,k}^*\right) \to \mathcal{N}\left(\boldsymbol{\delta}_k, \left(\boldsymbol{\mathcal{I}}_k^{*-1}\right)_{1,1}\right)$ follows from earlier arguments. Using this fact, we conclude that, under the sequence of local alternatives $H_{1,k}^\beta$, the adaptive de-biased deviance difference converges to a non-central $\chi^2$ random variable with $M^{(r)}$ degrees of freedom and non-centrality parameter $\nu_k^{(r)} := \boldsymbol{\delta}_k^\top \left(\boldsymbol{\mathcal{I}}_k^{*-1}\right)_{1,1} \boldsymbol{\delta}_k$:

$$\left[D_{k,\beta}^{(r)}\left(\hat{\boldsymbol{\omega}}_k^{(F)}, \hat{\boldsymbol{\omega}}_k^{(R)}\right) \middle| H_{1,k}^\beta\right] \to \chi^2(M^{(r)}, \nu_k^{(r)}). \tag{68}$$

□

## F.4 Proof of Corollary 2.1

*Proof.* Maximum-likelihood estimation of the parameters eliminates the need to de-bias the deviance, since the gradient evaluated at the maximum-likelihood estimate (and consequently, the bias terms) is exactly zero. Hence, the test statistic reduces to the adaptive deviance difference:

$$D_{k,\beta}^{(r)}\left(\hat{\boldsymbol{\mu}}_k^{(F)}, \hat{\boldsymbol{\mu}}_k^{(R)}\right) = \left(\frac{1+\beta}{1-\beta}\right) \left[2\left(\ell_k^\beta(\hat{\boldsymbol{\mu}}_k^{(F)}) - \ell_k^\beta(\hat{\boldsymbol{\mu}}_k^{(R)})\right)\right] \tag{69}$$

As in the course of proving Theorem 2, we begin with the deviance between the estimated and true parameters, $D_{k,\beta}\left(\hat{\boldsymbol{\mu}}_k, \boldsymbol{\mu}_k\right) = \left(\frac{1+\beta}{1-\beta}\right) \left[2\left(\ell_k^\beta(\hat{\boldsymbol{\mu}}_k^{(F)}) - \ell_k^\beta(\boldsymbol{\mu}_k)\right)\right]$. Noting that

$$\ell_k^\beta(\boldsymbol{\mu}_k) = \ell_k^\beta(\hat{\boldsymbol{\mu}}_k) + \frac{1}{2}(\hat{\boldsymbol{\mu}}_k - \boldsymbol{\mu}_k)^\top \nabla^2 \ell_k^\beta(\tilde{\boldsymbol{\mu}}_k)(\hat{\boldsymbol{\mu}}_k - \boldsymbol{\mu}_k), \tag{70}$$

the deviance can be expressed as

$$D_{k,\beta}\left(\hat{\boldsymbol{\mu}}_k, \boldsymbol{\mu}_k\right) = -\left(\frac{1+\beta}{1-\beta}\right) (\hat{\boldsymbol{\mu}}_k - \boldsymbol{\mu}_k)^\top \nabla^2 \ell_k^\beta(\boldsymbol{\mu}_k)(\hat{\boldsymbol{\mu}}_k - \boldsymbol{\mu}_k) + o_{\mathbb{P}}(1). \tag{71}$$

To proceed, we first argue that asymptotic results analogous to Propositions 1 and 2 hold. To determine the limiting behavior of the Hessian, note that it can be written as $\nabla^2 \ell_k^\beta(\boldsymbol{\mu}_k) = (1-\beta)\sum_{t=1}^k \beta^{k-t} \nabla^2 \ell_t(\boldsymbol{\mu}_k)$; evaluating both sides, it can be shown that $\nabla^2 \ell_k^\beta(\boldsymbol{\mu}_k) = \nabla^2 \ell_k(\boldsymbol{\mu}_k)$ and is not a function of the simultaneous spiking observations. Hence, $\nabla^2 \ell_k^\beta(\boldsymbol{\mu}_k) = \mathbb{E}\left[\nabla^2 \ell_k^\beta(\boldsymbol{\mu}_k)\right] = -\boldsymbol{\mathcal{I}}_k$. It also follows that $\nabla^2 \ell_k^\beta(\hat{\boldsymbol{\mu}}_k) = -\boldsymbol{\mathcal{I}}_k$ by the asymptotic consistency of $\hat{\boldsymbol{\mu}}_k$.

The normality of the gradient, i.e., that $\sqrt{\frac{1+\beta}{1-\beta}}\nabla\ell_k^\beta(\hat{\boldsymbol{\mu}}_k) \to \mathcal{N}(\mathbf{0}, \boldsymbol{\mathcal{I}}_k)$, is a consequence of Proposition 2. Note that, trivially, $\mathbb{E}\left[\nabla\ell_t(\hat{\boldsymbol{\mu}}_k)|\mathcal{F}_{t-1}\right] = 0$, where the filtration $\{\mathcal{F}_t\}_{t=0}^k$, as previously defined, corresponds to past spiking observations. Hence, the martingale CLT of Proposition 2 is applicable and the required conditions can be shown to hold. Alternatively, because $\nabla\ell_k^\beta(\hat{\boldsymbol{\mu}}_k)$ is the sum of independent but not identically distributed random variables, the Lindeberg-Feller CLT may be invoked to the same effect.

The limiting distribution of the adaptive deviance difference can now be established. First, we address the null hypothesis $H_{0,k} : \boldsymbol{\mu}_k^0 = (\boldsymbol{\mu}_{0,k}, \boldsymbol{\mu}_{1,k})$, where the parameters are partitioned into the free ($\boldsymbol{\mu}_{0,k}$) and restricted ($\boldsymbol{\mu}_{1,k}$) subsets as before. Through a series of arguments similar to those presented in proving Theorem 2, the limiting behaviors of $\nabla^2\ell_k^\beta(\boldsymbol{\mu}_k)$ and $\sqrt{\frac{1+\beta}{1-\beta}}\nabla\ell_k^\beta(\hat{\boldsymbol{\mu}}_k)$ imply that $D_{k,\beta}(\hat{\boldsymbol{\mu}}_k, \boldsymbol{\mu}_k) \to \chi^2(d)$, where $d$ is the dimensionality of the parameter vector; it likewise follows that the adaptive deviance difference is asymptotically $\chi^2$-distributed with $M^{(r)}$ degrees of freedom

$$\left[D_{k,\beta}^{(r)}\left(\hat{\boldsymbol{\mu}}_k^{(F)}, \hat{\boldsymbol{\mu}}_k^{(R)}\right)\middle|H_{0,k}\right] \to \chi^2(M^{(r)}), \tag{72}$$

where, as before, $M^{(r)}$ is the dimensionality of the subvector $\boldsymbol{\mu}_{1,k}$.

Next, we address the limiting distribution of the adaptive deviance difference under the sequence (in $\beta$) of local alternatives $\left\{H_{1,k}^\beta : \boldsymbol{\mu}_k^\beta = \left(\boldsymbol{\mu}_{0,k}^*, \boldsymbol{\mu}_{1,k} + \sqrt{\frac{1-\beta}{1+\beta}}\boldsymbol{\delta}\right)\right\}$, where $\beta$ converges to unity and $\boldsymbol{\mu}_k^*$ is the limiting true parameter vector. The deviances considered in this case are between the estimated and true limiting parameter, $D_{k,\beta}(\hat{\boldsymbol{\mu}}_k, \boldsymbol{\mu}_k^*)$.

The characterization of the limiting behavior of the Hessian is true under the sequence of alternatives, but the asymptotic normality of $\sqrt{\frac{1+\beta}{1-\beta}}\left(\hat{\boldsymbol{\mu}}_k - \boldsymbol{\mu}_k^*\right)$ must be established. To this end, we derive an equivalent expression based on the following Taylor expansions of the gradient at $\hat{\boldsymbol{\mu}}_k$ and $\boldsymbol{\mu}_k^\beta$ around $\boldsymbol{\mu}_k^*$:

$$\begin{aligned}\nabla\ell_k^\beta(\hat{\boldsymbol{\mu}}_k) &= \nabla\ell_k^\beta(\boldsymbol{\mu}_k^*) + \nabla^2\ell_k^\beta(\boldsymbol{\mu}_k^*)\left(\hat{\boldsymbol{\mu}}_k - \boldsymbol{\mu}_k^*\right) + o_{\mathbb{P}}(1-\beta)\\ \nabla\ell_k^\beta(\boldsymbol{\mu}_k^\beta) &= \nabla\ell_k^\beta(\boldsymbol{\mu}_k^*) + \nabla^2\ell_k^\beta(\boldsymbol{\mu}_k^*)\left(\boldsymbol{\mu}_k^\beta - \boldsymbol{\mu}_k^*\right) + o_{\mathbb{P}}(1-\beta).\end{aligned} \tag{73}$$

By rearranging the terms of these two equations, and by noting that $\nabla\ell_k^\beta(\hat{\boldsymbol{\mu}}_k) = 0$, the following equivalence is derived:

$$\begin{aligned}\hat{\boldsymbol{\mu}}_k - \boldsymbol{\mu}_k^* &= -\left(\nabla^2\ell_k^\beta(\boldsymbol{\mu}_k^*)\right)^{-1}\nabla\ell_k^\beta(\boldsymbol{\mu}_k^*) + o_{\mathbb{P}}(1-\beta)\\ &= \boldsymbol{\mu}_k^\beta - \boldsymbol{\mu}_k^* - \left(\nabla^2\ell_k^\beta(\boldsymbol{\mu}_k^*)\right)^{-1}\nabla\ell_k^\beta(\boldsymbol{\mu}_k^\beta) + o_{\mathbb{P}}(1-\beta).\end{aligned} \tag{74}$$

Denoting $\boldsymbol{\mu}_k^\beta - \boldsymbol{\mu}_k^*$ as $\tilde{\boldsymbol{\delta}}_k := \left[\mathbf{0}^\top, \sqrt{\frac{1-\beta}{1+\beta}}\boldsymbol{\delta}_k^\top\right]^\top$, it follows from the limiting behavior of $\nabla^2\ell_k^\beta(\boldsymbol{\mu}_k^*)$, the asymptotic normality of $\sqrt{\frac{1+\beta}{1-\beta}}\nabla\ell_k^\beta(\boldsymbol{\mu}_k^\beta)$ (by Proposition 2, or alternatively by the Lindeberg-Feller CLT), and by invoking Slutksy's theorem that $\sqrt{\frac{1+\beta}{1-\beta}}\left(\hat{\boldsymbol{\mu}}_k - \boldsymbol{\mu}_k^*\right) \to \mathcal{N}\left(\tilde{\boldsymbol{\delta}}_k, (\boldsymbol{\mathcal{I}}_k^*)^{-1}\right)$.

Recalling the partition of the parameter vector into subsets of free and restricted parameters under the null hypothesis and the corresponding decomposition of the Fisher information matrix $\boldsymbol{\mathcal{I}}_k^*$, the deviance difference between the full and reduced

models can be expressed as

$$D_{k,\beta}^{(r)}\left(\hat{\boldsymbol{\mu}}_k^{(F)}, \hat{\boldsymbol{\mu}}_k^{(R)}\right) = \left(\frac{1+\beta}{1-\beta}\right)\left(\hat{\boldsymbol{\mu}}_k^{(F)} - \boldsymbol{\mu}_k^*\right)^\top \boldsymbol{\mathcal{I}}_k^*\left(\hat{\boldsymbol{\mu}}_k^{(F)} - \boldsymbol{\mu}_k^*\right) - \left(\frac{1+\beta}{1-\beta}\right)\left(\hat{\boldsymbol{\mu}}_{0,k}^{(R)} - \boldsymbol{\mu}_{0,k}^*\right)^\top \boldsymbol{\mathcal{I}}_{0,0,k}^*\left(\hat{\boldsymbol{\mu}}_{0,k}^{(R)} - \boldsymbol{\mu}_{0,k}^*\right) + o_{\mathbb{P}}(1). \tag{75}$$

As before, we focus on the latter term. The first of the Taylor expansions in Eq. (73) implies that

$$\nabla \ell_k^\beta(\boldsymbol{\mu}_k^*) = \boldsymbol{\mathcal{I}}_k^*\left(\boldsymbol{\mu}_k - \hat{\boldsymbol{\mu}}_k^*\right) + o_{\mathbb{P}}(1),$$

and so the reduce model deviance can be written as

$$\left(\frac{1+\beta}{1-\beta}\right)\nabla_{\boldsymbol{\mu}_{0,k}}\ell_k^\beta(\boldsymbol{\mu}_k^*)^\top \boldsymbol{\mathcal{I}}_{0,0,k}^{-1}\nabla_{\boldsymbol{\mu}_{0,k}}\ell_k^\beta(\boldsymbol{\mu}_k^*) + o_{\mathbb{P}}(1). \tag{76}$$

Following a series of arguments analogous to those presented in the proof of Theorem 2, the deviance difference can be shown to be equivalent to

$$D_{k,\beta}^{(r)} = \left(\frac{1+\beta}{1-\beta}\right)\left(\hat{\boldsymbol{\mu}}_{1,k}^{(F)} - \boldsymbol{\mu}_{1,k}^*\right)^\top \tilde{\boldsymbol{\mathcal{I}}}_k^*\left(\hat{\boldsymbol{\mu}}_{1,k}^{(F)} - \boldsymbol{\mu}_{1,k}^*\right) + o_{\mathbb{P}}(1), \tag{77}$$

where $\tilde{\boldsymbol{\mathcal{I}}}_k^* := \boldsymbol{\mathcal{I}}_{1,1,k}^* - \boldsymbol{\mathcal{I}}_{1,0,k}^*\left(\boldsymbol{\mathcal{I}}_{0,0,k}^*\right)^{-1}\boldsymbol{\mathcal{I}}_{0,1,k}^* = \left(\boldsymbol{\mathcal{I}}_k^{*-1}\right)_{1,1}^{-1}$. That $\sqrt{\frac{1+\beta}{1-\beta}}\left(\hat{\boldsymbol{\mu}}_{1,k}^{(F)} - \boldsymbol{\mu}_{1,k}^*\right) \to \mathcal{N}\left(\boldsymbol{\delta}_k, \left(\boldsymbol{\mathcal{I}}_k^{*-1}\right)_{1,1}\right)$ follows from previous arguments. We thus conclude that, in the special case of the contemporaneous-event model, the adaptive deviance difference converges to a non-central $\chi^2$-distributed random variable with $M^{(r)}$ degrees of freedom and non-centrality parameter $\nu_k^{(r)} := \boldsymbol{\delta}_k^\top\left(\boldsymbol{\mathcal{I}}_k^{*-1}\right)_{1,1}\boldsymbol{\delta}_k$ under a sequence of local alternative hypotheses:

$$\left[D_{k,\beta}^{(r)}\left(\hat{\boldsymbol{\mu}}_k^{(F)}, \hat{\boldsymbol{\mu}}_k^{(R)}\right)\middle| H_{1,k}^\beta\right] \to \chi^2(M^{(r)}, \nu_k^{(r)}). \tag{78}$$

□

## F.5 Proof of Corollary 2.2

*Proof.* Recall from the proof of Theorem 2 that the partition of the parameter vector, $\boldsymbol{\omega}_k^0 = (\boldsymbol{\omega}_{0,k}, \boldsymbol{\omega}_{1,k})$, corresponds to the free parameters under the null hypothesis ($\boldsymbol{\omega}_{0,k}$) and the restricted sub-vector ($\boldsymbol{\omega}_{1,k}$), which are also free when estimating the full model for the alternative hypothesis. For the statistical inference of $r^{\text{th}}$-order coordination, $\boldsymbol{\omega}_{1,k}$ corresponded to the base rate parameters of $r^{\text{th}}$-order events; however, for the arbitrary nested hypotheses in Corollary 2.2, $\boldsymbol{\omega}_{1,k}$ corresponds to the arbitrary sub-vector that was restricted under the null hypothesis to estimate the reduced model. Denoting the limiting $\chi^2$ distributions' degrees of freedom by $M$ and the non-centrality parameter by $\nu_{(k)}$, the result is established, *mutatis mutandis*, by the same proof as of Theorem 2.

□

# References


1. van de Geer S, Buhlmann P, Ritov Y, Dezeure R. On asymptotically optimal confidence regions and tests for high-dimensional models. Ann Statist. 2014;42(3):1166–1202. doi:10.1214/14-AOS1221.

2. Daley DJ, Vere-Jones D. An Introduction to the Theory of Point Processes. vol. 1. 2nd ed. New York, NY: Springer; 2003.

3. Solo V. Likelihood functions for multivariate point processes with coincidences. In: Proceedings of IEEE Conference on Decision and Control. vol. 3; 2007. p. 4245 – 4250.

4. Kass RE, Kelly RC, Loh WL. Assessment of synchrony in multiple neural spike trains using loglinear point process models. Ann Appl Stat. 2011;5(2B):1262–1292. doi:10.1214/10-AOAS429.

5. Ba D, Temereanca S, Brown EN. Algorithms for the analysis of ensemble neural spiking activity using simultaneous-event multivariate point-process models. Frontiers in Computational Neuroscience. 2014;8(6). doi:10.3389/fncom.2014.00006.

6. ichi Amari S. Information geometry on hierarchy of probability distributions. IEEE Transactions on Information Theory. 2001;47(5):1701 – 1711. doi:10.1109/18.930911.

7. Schneidman E, Berry MJ, Segev R, Bialek W. Weak pairwise correlations imply strongly correlated network states in a neural population. Nature. 2006;440:1007–1012. doi:https://doi.org/10.1038/nature04701.

8. Ganmor E, Segev R, Schneidman E. Sparse low-order interaction network underlies a highly correlated and learnable neural population code. Proceedings of the National Academy of Sciences. 2011;108(23):9679–9684. doi:10.1073/pnas.1019641108.

9. Haykin SS. Adaptive Filter Theory. Upper Saddle River, NJ: Prentice Hall; 1996.

10. Mukherjee S, Babadi B. Dynamic Analysis of Higher-Order Coordination in Neuronal Assemblies via De-Sparsified Orthogonal Matching Pursuit. In: Advances in Neural Information Processing Systems. vol. 34; 2021. p. 4120–4133.

11. Zhang T. Sparse Recovery With Orthogonal Matching Pursuit Under RIP. IEEE Transactions on Information Theory. 2011;57(9):6215–6221. doi:10.1109/TIT.2011.2162263.

12. Kazemipour A, Wu M, Babadi B. Robust Estimation of Self-Exciting Generalized Linear Models With Application to Neuronal Modeling. IEEE Transactions on Signal Processing. 2017;65(12):3733–3748. doi:10.1109/TSP.2017.2690385.

13. Litwin-Kumar A, Harris KD, Axel R, Sompolinsky H, Abbott LF. Optimal Degrees of Synaptic Connectivity. Neuron. 2017;93(5):1153–1164.e7. doi:https://doi.org/10.1016/j.neuron.2017.01.030.

14. Pfau D, Pnevmatikakis EA, Paninski L. Robust learning of low-dimensional dynamics from large neural ensembles. In: Advances in Neural Information Processing Systems. vol. 26. Curran Associates, Inc.; 2013.

15. Thomson AM, Bannister AP. Interlaminar Connections in the Neocortex. Cerebral Cortex. 2003;13(1):5–14. doi:10.1093/cercor/13.1.5.

16. Sheikhattar A, Fritz JB, Shamma SA, Babadi B. Recursive Sparse Point Process Regression With Application to Spectrotemporal Receptive Field Plasticity Analysis. IEEE Transactions on Signal Processing. 2016;64(8):2026–2039. doi:10.1109/TSP.2015.2512560.

17. Mukherjee S, Babadi B. A Statistical Approach to Dynamic Synchrony Analysis of Neuronal Ensemble Spiking. In: Proceedings of the 2019 Asilomar Conference on Signals, Systems, and Computers, Nov. 3-6, Pacific Grove, CA; 2019.

18. Anderson BDO, Moore JB. Optimal Filtering. Courier Corporation; 2012.

19. Javanmard A, Montanari A. Confidence Intervals and Hypothesis Testing for High-Dimensional Regression. Journal of Machine Learning Research. 2014;15(82):2869–2909.

20. Zhang CH, Zhang SS. Confidence intervals for low dimensional parameters in high dimensional linear models. Journal of the Royal Statistical Society Series B (Statistical Methodology). 2014;76(1):217–242.

21. Needell D, Tropp JA. CoSaMP: Iterative signal recovery from incomplete and inaccurate samples. Applied and Computational Harmonic Analysis. 2009;26(3):301–321.

22. Davidson RR, Lever WE. The Limiting Distribution of the Likelihood Ratio Statistic under a Class of Local Alternatives. Sankhya: The Indian Journal of Statistics, Series A (1961-2002). 1970;32(2):209–224.

23. Sheikhattar A, Miran S, Liu J, Fritz JB, Shamma SA, Kanold PO, et al. Extracting neuronal functional network dynamics via adaptive Granger causality analysis. Proceedings of the National Academy of Sciences. 2018;115(17):E3869 – E3878. doi:10.1073/pnas.1718154115.

24. Crowder MJ. Maximum Likelihood Estimation for Dependent Observations. Journal of the Royal Statistical Society Series B (Methodological). 1976;38(1):45–53.

25. Brown BM. Martingale Central Limit Theorems. Annals of Mathematical Statistics. 1971;42(1):59–66. doi:10.1214/aoms/1177693494.

26. Wilks SS. The Large-Sample Distribution of the Likelihood Ratio for Testing Composite Hypotheses. Ann Math Statist. 1938;9(1):60–62. doi:10.1214/aoms/1177732360.

27. Wald A. Tests of Statistical Hypotheses Concerning Several Parameters When the Number of Observations is Large. Transactions of the American Mathematical Society. 1943;54(3):426–482. doi:10.2307/1990256.
