# Supplementary material for: Adaptive modeling and inference of higher-order coordination in neuronal assemblies: A dynamic greedy estimation approach
Source: PLoS Comput Biol. 2024 May 28;20(5):e1011605. doi: 10.1371/journal.pcbi.1011605 (PMC11161120; doi:10.1371/journal.pcbi.1011605)
Supplement: S2 Appendix — In this appendix, we present supporting simulations that address hyperparameter selection and the scalability of the proposed method to large neuronal assemblies. (PDF) [file pcbi.1011605.s002.pdf]

S2 Appendix
Supporting Simulations
for "Adaptive modeling and inference of higher-order coordination in neuronal assemblies: a dynamic greedy estimation approach"

Shoutik Mukherjee[1,2], Behtash Babadi[1,2*]

1 Department of Electrical and Computer Engineering, University of Maryland, College Park, MD, USA
2 Institute for Systems Research, University of Maryland, College Park, MD, USA

* behtash@umd.edu

Overview. In this appendix, we present supporting simulations that first address hyperparameter selection and then the scalability of the proposed method to large neuronal assemblies. We first demonstrate the effects of varying bin widths, the effective integration window through W and β, and the history integration window on the analysis of simulated ensemble spiking. Next, we analyzed the simulated ensemble spiking of 100 neurons to demonstrate applicability of the proposed methods to large populations of neurons.

Hyperparameter Selection

Bin Size

First, we consider the effect of the bin size used to discretize spiking observations in the context of the first simulated example (Fig 3). Spikes were generated as a discrete time process, so a smaller bin size would equate to upsampled spike trains, extending the number of frames between spikes without gaining or losing temporal resolution. However, increasing the bin size results in the coassignment of multiple spikes to the same bin; as illustrated in Fig S1, the consequent loss of temporal resolution distorts the underlying correlational structure of ensemble spiking.

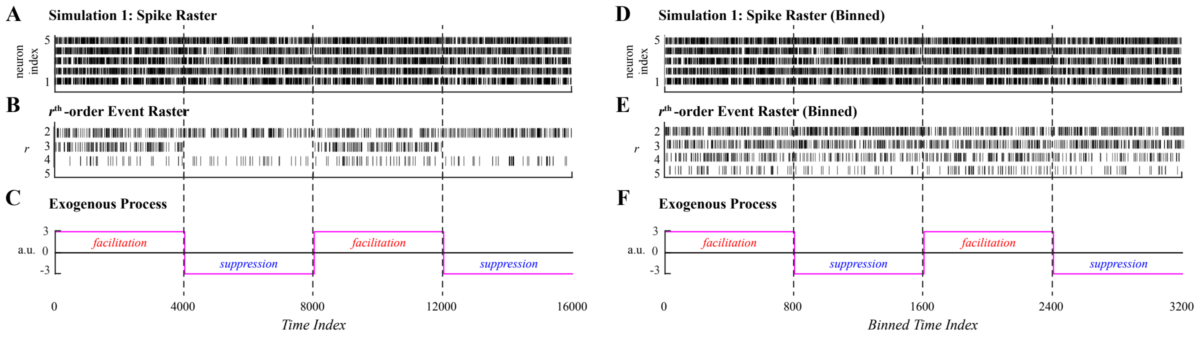

Fig S1. Large bin sizes results in loss of temporal resolution, distorting underlying correlational structure.

A bin size of 5 samples was used to downsample the generated spiking activity. While the spike rasters before and after binning (Figs S1**A** and S1**D**, respectively) do not obviously reflect distorted correlational structure, it is clear from the rth-order event rasters. In Fig S1**B**, the rate of 3rd-order simultaneous spiking modulates in consort with the exogenous process (Fig S1**C**). However, after binning, the rate of 3rd-order events no longer corresponds to the exogenous process (Figs S1**E** and S1**F**); concurrently, more 4th-order events are observed and 5th-order events, which were entirely absent from simulated activity, can be observed as well (Fig S1**E**).

The selection of the bin size thus constitutes an assumption about the temporal precision with which spikes can be considered simultaneous. To mitigate artifacts such as those illustrated in Fig S1, bin sizes should be chosen so that, for each neuron, the coassigment multiple spike times to the same bin is minimized. This criterion was employed in the selection of bin sizes for both the human anesthesia and rate sleep cycle data.

Effective Integration Window: W and β

Next, we consider the selection of hyperparameters that affected the timescales over which higher-order spiking coordination could be inferred, namely the window size W over which parameters are assumes constant and the forgetting factor β. The effective integration window length of the RLS-like algorithm utilized here was established to be $N_{\text{eff}} = \mathcal{O}\left(\frac{W}{1-\beta}\right)$ in [1]. Hence, the choice of large W or $\beta \approx 1$ imposes that the estimated time-varying model parameters evolve slowly, resulting in inferred patterns of higher-order coordination that emerge and persist over slower timescales.

In order to obtain a suitable combination (W, β) for each simulated example and gain insight into how the selection related to the simulated latent states, the window size over which parameters were assumed constant was set as $W = 10$ in order to enable stable estimation of the MkPP model at each window while still allowing for fast

changes. Goodness-of-fit was evaluated using graphical Kolmogorov-Smirnoff (KS) and autocorrelation function (ACF) tests of the mark CIFs, invoking the multivariate generalization of the time-rescaling theorem for point processes [2,3]. The KS test plots empirical conditional density function (CDF) of time-rescaled interspike intervals against uniform quantiles to determine if they are uniformly distributed; and the ACF test determines if the interspike intervals are uncorrelated.

We inspected the KS and ACF tests of the 1st-order events to assess if MkPP models were adequately well-estimated. The occurrence of 1st-order events with greater frequency than higher-order events translates to more samples with which to construct empirical distributions of interspike intervals, and consequently tighter confidence intervals. In Fig S2, we compare the KS and ACF tests of the same 1st-order event when a MkPP model was fit to simulated spiking data using $W = 10, 5, 20$ (Fig S2**A**–**C**, respectively); the forgetting factor was fixed to $\beta = 0.975$. The KS and ACF tests in Fig S2**A**–**C** are shown with 95% confidence intervals (red).

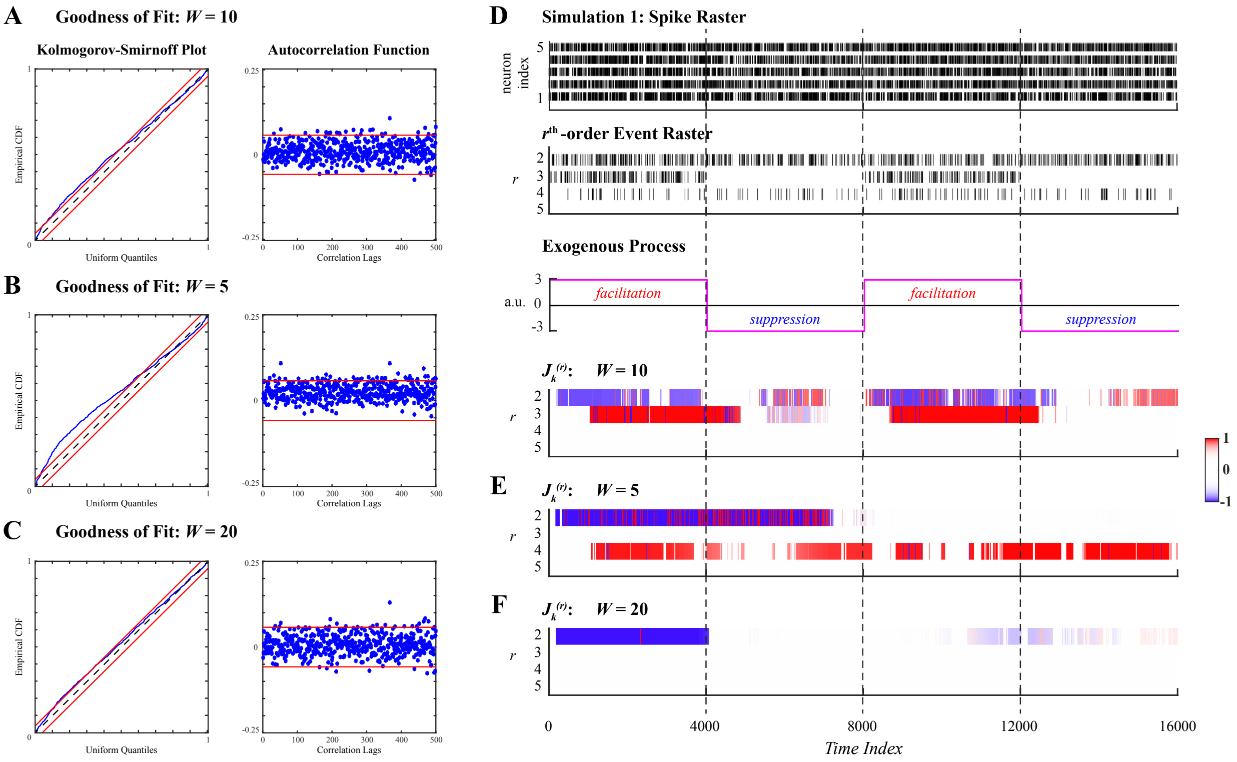

Fig S2. Effects of varying W on model goodness-of-fit and history-dependent inference of higher-order coordination. **A**–**C.** Graphical Kolmogorov-Smirnoff (KS; left) and autocorrelation function (ACF; right) tests for goodness-of-fit. Dashed black line in KS plot indicates exact match between the empirical cumulative density function (CDF) and CDF of a uniform distribition. Red lines indicate the 95% confidence interval. **D.** History-dependent analysis of simulated example 1 with $W = 10$, reproduced from Fig 3. **E.** History-dependent analysis with $W = 5$. **F.** History-dependent analysis with $W = 20$.

The combination $(W, \beta) = (10, 0.975)$, which was used in the analysis reported in Fig 3 of the main text, produced an estimated MkPP model in which the time-rescaled intervals between 1st-order events are uniformly distributed and uncorrelated (Fig S2**A**). Decreasing the window length to $W = 5$ introduced a mismatch in the lower quantiles of the distribution of time-rescaled interspike intervals and a small positive bias in the autocorrelation function (Fig S2**B**), indicating comparatively poor model fit. Additionally, a smaller W reduces the effective integration window so that the model

evolves faster over time; consequently, faster history-dependent dynamics in 4th-order dynamics are misidentified as significantly coordinated while the slower exogenously driven 3rd-order are missed (Fig S2**E**). Increasing the window length to $W = 20$ resulted in similar model goodness-of-fit as $W = 10$ (Fig S2**C**), but a longer effective integration window so that the model evolved more slowly; hence, no significant 3rd-order coordination was detected (Fig S2**F**).

Having fixed W, several candidate values for β were considered to obtain the most appropriate effective integration window. Representative examples of history-dependent analysis of higher-order coordination in both simulated examples using different forgetting factors are shown in Fig S3. Analyses of the first simulated data set are shown in Fig S3**A**–**G**, and the second simulated data set in Fig S3**H**–**N**. Here, both simulations were examined in order to demonstrate the efficacy of the heuristic for choosing β, outlined in the following. The simulated data, r^{th}-order event raster plots, exogenous processes, and history-dependent analysis results from the main text are provided for reference (Fig S3**A**–**D** and S3**H**–**K**).

For practical purposes, we approximated $N_{\text{eff}} \approx \frac{W}{1-\beta}$, and examined the relationship between N_{eff} and the duration of latent states in the simulated data; we denote the duration of shortest latent state by τ. The best choices of β (Fig S3**D** and S3**K**), determined by how closely inferred higher-coordination corresponded to the ground-truth latent dynamics, were those for which $N_{\text{eff}} \approx \frac{\tau}{10}$. The square wave in the first simulation had half-periods of $\tau = 4000$ samples that determined if the exogenous effect on 3rd-order coordination was facilitated or suppressed; for $\beta = 0.975$, $N_{\text{eff}} = 400$. In the second simulation, we noted that the exogenous autoregressive process most persistently facilitated 3rd-order coordinated spiking for a duration of ~ 4000 samples. Within that duration, two subintervals of $\tau \approx 2000$ samples separated at time index ~ 8000 could be discerned upon visual inspection (Fig S3**J**); for $\beta = 0.95$, $N_{\text{eff}} = 200$.

We validated the choices of τ for each simulated data set by performing higher-order

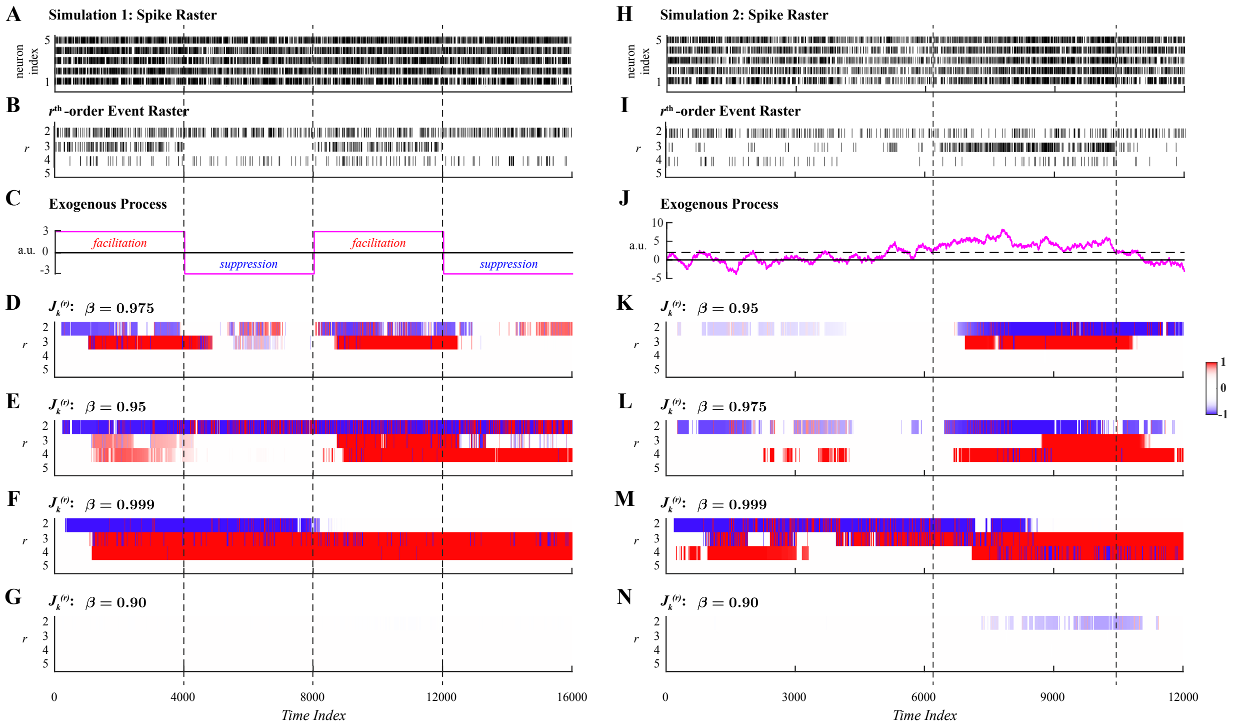

Fig S3. Effects of varying β in history-dependent analysis of simulated data.

coordination analysis with the best choice of β for the other data set. That is, we used $\beta = 0.95$ to analyze the first simulated data set instead of $\beta = 0.975$, and vice versa for the second simulation. The results (Fig S3**E** and S3**L**) prominently indicated false detection of 4th-order exogneous coordination. Additionally, 3rd-order coordination was identified transiently and with less statistical strength during the first facilitative half-period (Fig S3**E**) of the first simulation. Meanwhile, there was a pronounced delay between the onset of facilitated 3rd-order coordinated spiking and its detection in the second simulation (Fig S3**L**). These are characteristic effects of smaller or larger choices of β, respectively. To illustrate the effect more clearly, history-dependent higher-order coordination analysis was applied to each simulated data set with representative examples of large and small β. Specifically, results of the analysis using $\beta = 0.999$ (Fig S3**F** and S3**M**) and $\beta = 0.9$ (Fig S3**G** and S3**N**) are shown. Using a large forgetting factor that corresponded to $N_{\text{eff}} = 10000$ resulted in slower dynamics, as indicated by insensitivty to fast state transitions and the false detection of 4th-order exogenous coordination. In contrast, using a small forgetting factor that corresponded to $N_{\text{eff}} = 100$ resulted in estimated models adapting to fast variations in spiking statistics and a subsequent inability to track slower latent dynamics.

Although choosing β so that $N_{\text{eff}} \approx \frac{\tau}{10}$ seemed a viable criterion for the simulated data, the analyses of recorded data sets exhibited significant coordination more transiently and with less statistical strength, consistent with an effective integration window too small to track transitions between salient latent states. These issues in identifying robust statistically significant coordination were resolved by choosing β so that $N_{\text{eff}} \approx \frac{\tau}{5}$. Unlike the recorded data sets, the simulated data sets had more uniformity amongst the neuronal assembly. Specifically, simulated neurons had similar firing rate dynamics because the mark CIFs of all r^{th}-order marked events were equal. For example, all 3rd-order events had the same probability as each other but not necessarily the same probability as 2nd- or 4th-order events. This uniformity likely enabled latent dynamics to be accurately inferred using a shorter effective integration window than was necessary for recorded ensemble spiking.

Parameterization of History-Dependence

Finally, we address the effect of the length of history-dependence through the parameterization of the history integration window. The generating models for simulated data and the results analyzing simulated and physiological data all used the same history integration window spanning the $p = 15$ time lags. To control model complexity, the history integration window was subdivided into $M = 4$ non-overlapping windows with lengths $\{P_m\}_{m=1}^{M} = \{2^{m-1}\}_{m=1}^{M}$; rather than fitting 15 coefficients to capture the effect of a neuron's spiking history on one mark, this approach only required fitting 4. To demonstrate the effect of varying the length of history-dependence, the first simulated data set was analyzed using history integration windows consisting of $M = 3$ subintervals (equivalently, $p = 7$ time lags) and of $M = 5$ subintervals ($p = 31$ time lags); the inference of higher-order spiking coordination under each choice of M is shown in Fig S4. The window length and forgetting factor were set to $(W, \beta) = (10, 0.975)$ in all cases.

When the history integration window was half the length of the true window, the underlying exogenous facilitation of 3rd-order events was not detected because of model mismatch (Fig S4**A** and S4**B**). Specifically, the common history-related effects not accounted for by the short history integration window in both the full and reduced models introduced a confound that hindered the ability to detect the exogenous process' influence. In contrast, when the integration window was double the true size, the analysis correctly identified exogenous coordination of 3rd-order events in the correct epochs of the simulation (Fig S4**A** vs. Fig S4**C**). This indicates that it is preferable to

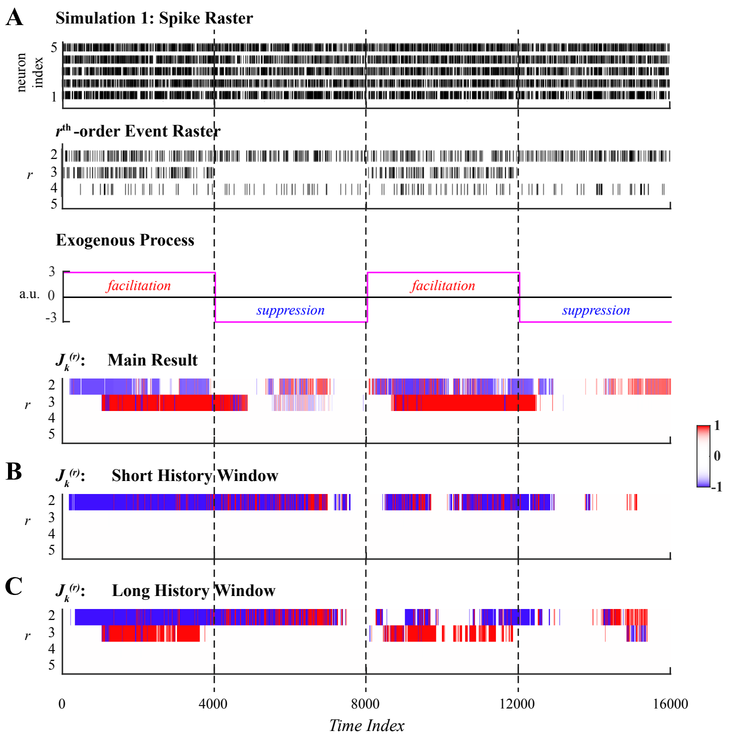

Fig S4. Effects of varying length of history-dependence on analysis of simulated data.

overparameterize the MkPP model for history-dependent analysis, allowing greedy support selection to identify the most relevant covariates.

Application to Large Neuronal Assemblies

To demonstrate the applicability of higher-order coordinated spiking analysis in large neuronal assemblies, we considered an additional simulated assembly of 100 neurons. The spiking activity (Fig S5**A**) was simulated so that during the first 1000 samples, no exogenous coordination or endogenous synchronization occurred. However, a subset of 3rd-order events were exogenously induced during the second 1000 samples of the simulation (Fig S5**B**).

Before pruning, the mark space has cardinality of $2^{100} - 1$ possible simultaneous spiking events. After identifying the reliable interactions with a threshold $N_{thr} = 1$, the mark space had a cardinality of 5100 events, consisting of all 100 1st-order events, all 4950 2nd-order events, and 50 reliably occurring 3rd-order events. Applying the history-dependent analysis with hyperparameters $(W, \beta) = (10, 0.975)$, the exogenous coordination of 3rd-order events was accurately recovered (Fig S5**C**). This simulation thus demonstrates that the proposed adaptive marked point process estimation and statistical inference procedures, including appropriately pruning the mark space, can be used to identify higher-order coordination in large neuronal assemblies.

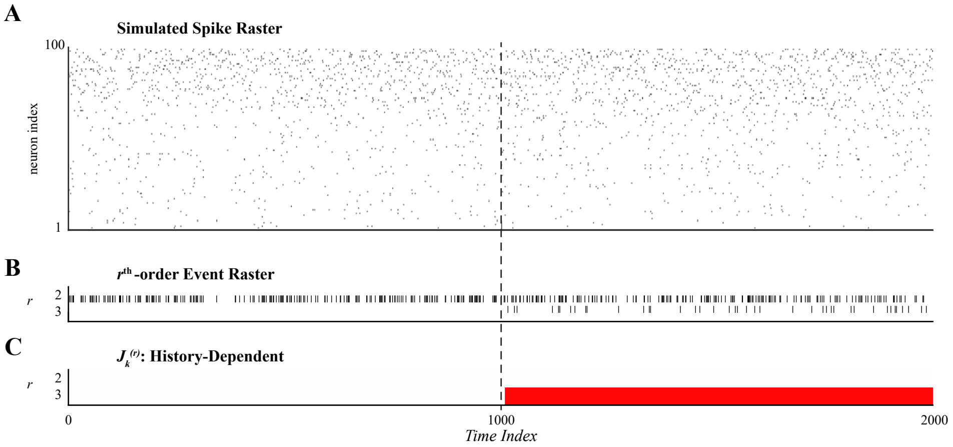

Fig S5. History-dependent analysis of higher-order coordinated spiking in an assembly of 100 neurons. **A.** Simulated spiking activity of 100 neurons with no exogenous coordination for the first half of the simulated duration and exogenously induced 3rd-order interactions during the second half of the simulated duration. **B.** Latent dynamics of higher-order coordinated spiking were evident in raster of rth-order spiking events. **C.** Exogenous coordination was correctly detected using the history-dependent model of the marked point process.

References

1. Sheikhattar A, Fritz JB, Shamma SA, Babadi B. Recursive Sparse Point Process Regression With Application to Spectrotemporal Receptive Field Plasticity Analysis. IEEE Transactions on Signal Processing. 2016;64(8):2026–2039. doi:10.1109/TSP.2015.2512560.

2. Ba D, Temereanca S, Brown EN. Algorithms for the analysis of ensemble neural spiking activity using simultaneous-event multivariate point-process models. Frontiers in Computational Neuroscience. 2014;8(6). doi:10.3389/fncom.2014.00006.

3. Daley DJ, Vere-Jones D. An Introduction to the Theory of Point Processes. vol. 1. 2nd ed. New York, NY: Springer; 2003.
