# Supplementary material for: Adaptive modeling and inference of higher-order coordination in neuronal assemblies: A dynamic greedy estimation approach
Source: PLoS Comput Biol. 2024 May 28;20(5):e1011605. doi: 10.1371/journal.pcbi.1011605 (PMC11161120; doi:10.1371/journal.pcbi.1011605)
Supplement: Attachment — Submitted filename: MukherjeeBabadi_PLoSCompBiol_ReviewerResponses.pdf [file pcbi.1011605.s003.pdf]

Responses to Reviewer Comments on "Adaptive modeling and inference of higher-order coordination in neuronal assemblies: a dynamic greedy approach" (PCOMPBIOL-D-23-01661)

Shoutik Mukherjee and Behtash Babadi
Department of Electrical & Computer Engineering
Institute for Systems Research
University of Maryland, College Park, MD 20742, USA

Preface

We thank the reviewers and reviewing editors for their thoughtful feedback on our manuscript. We have now substantially revised the manuscript by including additional simulations to characterize the interplay of key hyperparameters, and by improving the contextualization of our contributions and the accessibility of our presentation to a broader audience. In summary, the major changes include:

1) A new subsection under **Methods**, namely **Related Work**, that specifically compares the proposed method to two closely-related model-based approaches as well as model-free measures of synchrony.
2) The reduction of mathematical details in the main text that are superfluous to intuitive explanations of the proposed methods, and their addition to the revised supporting information file **S1 Appendix** that outlines algorithm development and theoretical results.
3) The addition of more simulations addressing the choice of bin size, history integration window length, and size of windows over which parameters are constant to complement existing simulations addressing the choice of the forgetting factor. All simulations addressing hyperparameter selection have been combined and presented in the newly added supporting information file, **S2 Appendix**.
4) Clarifications of the relevance that the scientific insights gained by applying the proposed methods to physiological data have for neuroscientists provided in the **Discussion**.

In the following, we respond (in black) point-by-point to each of the reviewers' comments (in green). We have included identical versions of the main text and S1 Appendix that highlight changes (in red) for ease of tracking.

Response to the Editor's Comments

In particular, the authors should ensure that the relationship between their method and previous, related algorithms is well described, to provide better context for the novel contribution made here; and that the mathematical details are complemented by a more intuitive description of their approach which will increase the impact of this manuscript by making it more accessible, particularly to experimental neuroscientists. To that end, it may even be worth moving some

mathematical details to the supplementary information, if they feel that this could improve the readability of the paper.

Response: We thank the reviewers and editors for their comments and feedback to improve the accessibility of the proposed methods and results. Our manuscript has been substantially revised to emphasize the intuition of our approach and its relation to existing approaches, and reorganizing the methods and mathematical details to make the paper more accessible for the PLoS Comp. Biol. audience and particularly to experimental neuroscientists. Below, we respond to each of the reviewers' specific comments.

Response to the Comments of Reviewer #1

Reviewer #1:
The authors propose a novel method to detect significant high-order correlations in multi-neuron spike trains in the absence of trial structure. The methodology appears rigorous, although the mathematical details are quite dense and not possible for me to exhaustively verify. The scientific significance of these findings is not communicated clearly, and the paper should include more head-to-head comparisons with baseline models.

Below I comment on the elements of the manuscript laid out in the reviewer guidelines.

Response: We thank the reviewer for their detailed suggestions to improve the clarity of our proposed methodology, their relation to existing statistical methods, and the results of their application to experimental data. Below, we have describe our revisions that address the reviewer's comments.

ORIGINALITY + INNOVATION. The method seems original, but is not directly compared to previous methods on simulated or real data in a head-to-head manner. The authors argue that previous approaches "require multiple repeated trials to capture dynamics in correlational structure." But it isn't immediately clear why this is true, particularly since one of the baseline methods they mention is a state space model -- most state space models can be fit on unstructured time series data. I think at minimum there needs to be a section where the authors write down equations of prior models and explain why those models require multiple trials. It would be even better if the authors formulate some naive extensions of these existing models to the trial-free data and show how those naive extensions fail, whereas their approach succeeds. Currently, the paper is not self-contained, making it difficult for a reader to digest without first reading a long and niche sub-literature.

Response: We appreciate the reviewer's suggestions to improve the accessibility of the paper through additional contextualization of the proposed methods. The revised manuscript includes a section (Related Work, under Materials and Methods) that evaluates the formulations the model-based analyses highlighted, as well as the model-free information theoretic analyses that

Reviewer 2 has noted. Multiple trials are required to capture dynamics with statistical confidence when using the state-space or log-linear point process models that we note are closely related to the proposed method.

First, considering the maximum-entropy model in (Shimazaki et al., PLoS Comp. Biol., 2012) that is fit using Bayesian state-space filtering, within-trial non-stationarity and across-trial stationarity are assumed. The evolution of instantaneous correlation coefficients was modeled as a state transition, and an expectation-maximization (EM) algorithm is used to estimate these coefficients by maximizing the posterior density. The posterior, which is also utilized to construct credible intervals for the estimates, is formulated in terms of the instantaneous rate, for which the trial-averaged activity is a sufficient statistic. In the single-trial case assuming non-stationarity, the posterior would be poorly estimated.

Second, the log-linear point process model in (Kass, et al., Ann. Appl. Stat., 2011) tests if the rate of a simultaneous spiking event is explained by the product of the rates of each neuron by assessing if a multiplicative factor significantly diverges from 1 using bootstrapping. Stated explicitly for a two-neuron example, the log-probability of their simultaneous spiking is modeled as $\log P[n_1 = 1, n_2 = 1] = \log P[n_1 = 1] + \log P[n_2 = 1] + C$, and the null hypothesis that $C = 0$ is tested using bootstrapping. In order to capture time-varying latent coordination, constructing the bootstrap distribution for the factor C would require multiple repeated trials.

- *To address this comment of the reviewer, we have now added an entirely new section in the Methods, called "Related Work", which provides details of the existing relevant work and clarifies how existing methods cannot operate with single trial data (Pages 3-5).*

IMPORTANCE TO RESEARCHERS. The authors cite a number prior works that have developed similar statistical methodologies. The first paragraph of the introduction also mentions some motivations for the analysis from experimental neuroscience, but these are not really developed. Insights derived from experimental analysis could also be communicated more clearly -- for example, the authors observe changes in correlation structure associated with loss of consciousness, but it is not clear whether this could be used for a clinically relevant purpose or if simpler methods would not be sufficient for this application.

Response: We thank the reviewer for highlighting the clarity of the experimental analyses as a point for improvement. We concur that the insights into the anesthesia data set are attainable through other methods; the purpose of this application was in fact to empirically validate our method.

We directly compared previous analyses of this data (Lewis et al., PNAS, 2012) to our methods to confirm they can provide biologically plausible insights into localized correlates of brain state dynamics. Previous studies of anesthesia, including analysis of the data set in question, have indicated disruptions to global connectivity but not local connectivity under anesthesia; this is

directly corroborates our comparisons of the history-independent and history-dependent analyses that indicate no exogenous coordination after loss of consciousness.

Regarding the analyses of rat cortical neurons during a sleep cycle, first we note that state transitions during sleep have been typically characterized in terms of multiband analyses of electrophysiological data; and to the best of our knowledge, the properties of neuronal spiking have largely been considered in terms of non-parametric measures such as correlations, mean activity, Fano Factors, etc. The spiking dynamics in a simultaneously recorded population of neurons over the course of a sleep cycle hence appear not to be well-explored.

While consequently not being able to corroborate the results obtained from the rate cortical spiking dataset as extensively as the anesthesia dataset, we do note that the exogenous coordination of inhibitory neurons during REM sleep observed in our analysis are consistent with previous results that indicate signatures of REM sleep can be detected subcortically before changes in cortical activity are observed. Moreover, empirical validation via the anesthesia data gives credence to interpreting the analyses rat neuronal activity during a single sleep cycle, albeit cautiously, and motivates future studies of spiking dynamics during fast brain state transitions; this serves as one concrete instance of the relevance of the proposed methodology to experimentalists.

- *To address this comment of the reviewer, the revised manuscript more clearly outlines this empirical validation in the Discussion section, to clarifying the relevance of our results (Page 21).*

SIGNIFICANT BIOLOGICAL OR METHODOLOGICAL INSIGHT. As explained above I think the biological insights are weak and speculative. But there are clearly methodological insights and innovations in this paper.

Response: We appreciate the reviewer's suggestions to better contextualize the results obtained using the proposed method and have addressed these specifically in response to their comments under "originality+ innovation".

RIGOROUS METHODOLOGY. The methodology is complex and clearly the authors have put a lot of thought into it. They verify that the method behaves as expected on simulated data. On the other hand, there are elements of the method that appear to be ad hoc or heuristic. For example, the authors threshold a subset of the mark space with a parameter (N_thr) on page 6. It is clear why this is necessary for computational reasons, but it is less clear how users should set this value in practice, particularly since the number of thresholded marks will change as a function of the length of the recording. Does this thresholding introduce bias into the confidence intervals or statistical tests?

Response: We thank the reviewer for their detailed suggestions to clarify elements of the proposed methodology. The revised manuscript includes text revisions that clarify the

methodology and augmented simulation results that address the effect of hyperparameter selection on model goodness-of-fit.

First, we address the threshold parameter, N_{thr}. Because fitting the marked point process (MkPP) model essentially amounts to fitting a time-varying categorical distribution, pruning marks on the basis of the threshold criterion equates to limiting the number of categories to those that occur with non-negligible frequency.

What constitutes a negligible frequency is context dependent. In simulations, we choose a conservative value of $N_{thr} = 1$; that is, we treated all simultaneous spiking events that occurred at least once as non-negligible. Generally, an appropriate value for N_{thr} could be obtained in relation to the rate of simultaneous spiking events. In applications to experimental data, we provided spiking rates (in Hz and spikes per time bin) to serve as guidelines for a range of rates that could be used to set the threshold.

Because negligible events are excluded from the joint distribution, their rates are not modeled. This does not affect the construction of confidence intervals around estimated parameters or the statistical tests, which hold as described for a MkPP model with an arbitrarily large mark space.

Similarly, the window length W and forgetting factor beta seem like very important parameters that need to be tuned. The authors seem to have an intuition for how to pick these parameters, but it isn't spelled out clearly for the reader. For instance, on page 13 say they fix W to an intermediate value to trade off between "stable estimation... while still allow for fast changes." How should one quantify stable estimation? In Figure 3E, there is often flickering between red and blue, which would seem to be unstable predictions?

Response: The stability of estimated MkPP models was judged on the basis of graphical Kolmogorov-Smirnoff and autocorrelation function tests, which the multivariate extension of the time rescaling theorem states can be used to assess the goodness-of-fit of each mark's conditional intensity function in a similar manner as the univariate theorem for point process models (Ba et al., Front. Comput. Neurosci, 2014; Daley and Vere-Jones, 2003).

We have now added an entirely new appendix, namely **S2 Appendix**, containing several new numerical validations to demonstrate the effect of varying W on model goodness-of-fit, as well as the effect of bin width and the history integration window that Reviewer 2 has recommended.

Here, we reproduce Fig. S2 from **S2 Appendix** that shows the effect of changing W. In short, the combination $(W, \beta) = (10, 0.975)$, which was used in the analysis reported in Fig. 3 of the main text, produced an estimated MkPP model in which the time-rescaled intervals between 1st-order events are uniformly distributed and uncorrelated (Fig. S2A). Decreasing the window length to $W = 5$ introduced a mismatch in the lower quantiles of the distribution of time-rescaled interspike intervals and a small positive bias in the autocorrelation function (Fig. S2B), indicating comparatively poor model fit. Additionally, a smaller W reduces the effective integration window so that the model evolves faster over time; consequently, faster history-dependent dynamics in

4th-order dynamics are misidentified as significantly coordinated while the slower exogenously driven 3rd-order are missed (Fig. S2E). Increasing the window length to W = 20 resulted in similar model goodness-of-fit as $W = 10$ (Fig. S2C), but a longer effective integration window so that the model evolved more slowly; hence, no significant 3rd-order coordination was detected (Fig. S2F).

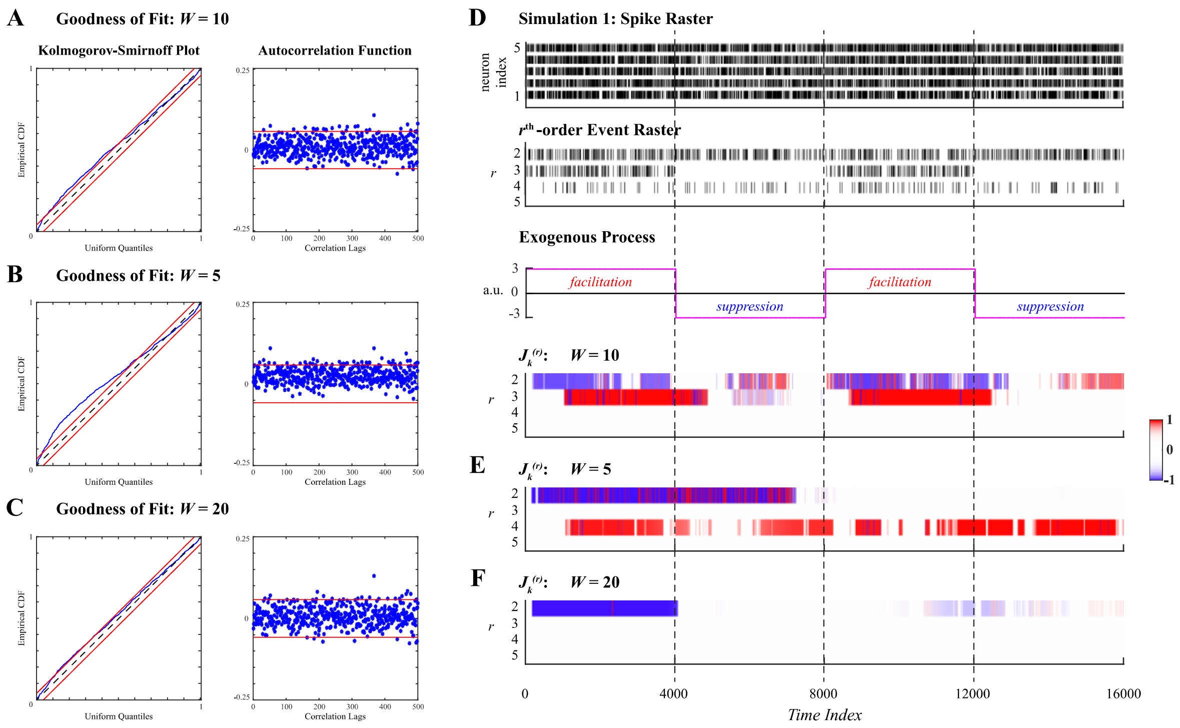

Fig S2. Effects of varying W on model goodness-of-fit and history-dependent inference of higher-order coordination. **A–C.** Graphical Kolmogorov-Smirnoff (KS; left) and autocorrelation function (ACF; right) tests for goodness-of-fit. Dashed black line in KS plot indicates exact match between the empirical cumulative density function (CDF) and CDF of a uniform distribition. Red lines indicate the 95% confidence interval. **D.** History-dependent analysis of simulated example 1 with $W = 10$, reproduced from Fig. 3. **E.** History-dependent analysis with $W = 5$. **F.** History-dependent analysis with $W = 20$.

- *To address this comment of the reviewer, we have now added an entirely new appendix, namely **S2 Appendix**, containing several new numerical validations to demonstrate the effect of varying W on model goodness-of-fit, as well as the effect of bin width and the history integration window that Reviewer 2 has recommended.*

After fixing W, the authors perform a sweep over the forgetting factor beta to achieve "the most appropriate value." What exactly is being optimized here? It seems like if these parameters are chosen incorrectly it will lead to a loss of statistical power, while on the other hand if they are optimized to create low p-values it would inflate type-I errors. I unfortunately cannot read supporting information S1 Appendix which seems important to these issues. Was it uploaded?

Response: The forgetting factor was selected so that the effect data length window, $N_{eff} = O(W/(1-\beta))$, corresponded to a fraction of the length of the shortest-duration simulated state,

which were defined by the simulated dynamics. The most appropriate choice of N_{eff} was determined based on the alignment of detected intervals significant higher-order coordination and the simulated state dynamics. Simulations, previously presented in S1 Appendix but now grouped with other supplementary simulations in **S2 Appendix**, demonstrate the efficacy of this heuristic (See below, Fig. S3 reproduced from **S2 Appendix**).

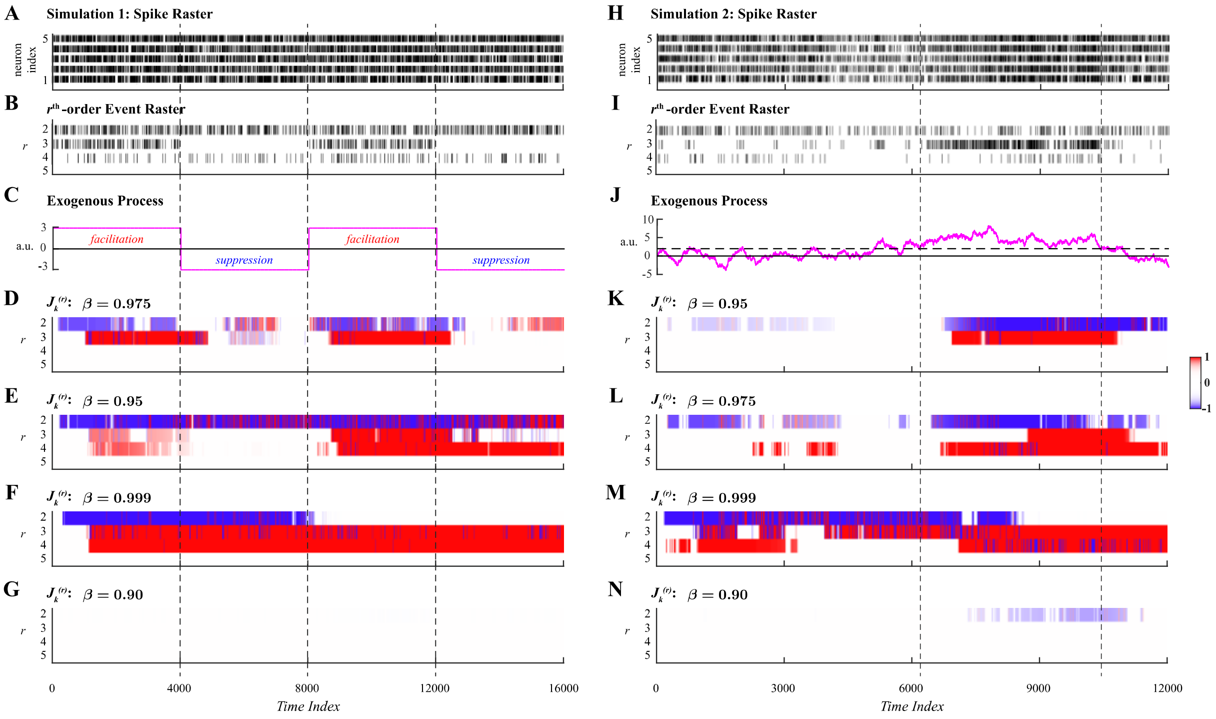

Fig S3. Effects of varying β in history-dependent analysis of simulated data.

- *To address this comment of the reviewer, we have now reported numerical experiments pertaining to the role of N_{eff} in **S2 Appendix** (Fig. S3) and have discussed its impact on the results.*

SUBSTANTIAL EVIDENCE FOR CONCLUSIONS. The point of this paper is to develop a statistical methodology and not to develop a scientific theory or test specific hypotheses (as far as I can tell). So this point does not seem particularly applicable. However, as mentioned above under "originality + innovation" I would like to see more clear discussions and comparisons with existing statistical methods. Ideally the authors should show how their method outperforms others head-to-head on simulated datasets.

Response: The reviewer correctly describes the main objective of this paper, and we appreciate the reviewer's suggestions to better contextualize the proposed method. We have addressed the revisions made to do so specifically in response to their comments under "originality+ innovation".

Response to the Comments of Reviewer #2

Reviewer #2:
This work proposes a well-substantiated methodology to assess higher-order coordination in neural spiking activity, as well as a framework for accurate statistical inference to rule out occurrences of random simultaneous spiking events. The methodology is validated in simulated settings and applied to neural spike train recordings of human and rat cortical assemblies. The experimental results are interesting, revealing the onset of different higher-order coordination patterns distinguishing consciousness from anesthesia in the human data, and sleep state transitions in the animal data. The paper is accurately written, though resulting rather difficult to follow for non-expert readers especially in the methodological description. A better placing in the context of the existing literature and description of the dependence on crucial parameters are suggested.

Response: We thank the reviewer for their suggestions to clarify the proposed method's relation to the existing literature and outline its dependence on hyperparameter selection, as well as to provide more intuitive descriptions of the methodology. We have outlined the revisions addressing the reviewer's specific comments below.

Specific comments:

1) The proposed method is based on statistical model representations, which are contrasted to more general model-free approaches only briefly in the introduction. Nevertheless, an emerging line of research for the model-free analysis of multiple spike trains, stemming from the methodological and applicative works listed below [R1-R4], seems overlooked in the present paper. These works, showing the feasibility and usefulness of multivariate model-free analysis of causal (history-dependent) interactions and synchronization in spike trains, should be acknowledged, and compared – at least at the level of discussion – with the statistical models on which the present contribution is grounded.
   [R1] Shorten, D. P., et al. (2021). Estimating transfer entropy in continuous time between neural spike trains or other event-based data. PLoS computational biology, 17(4), e1008054.
   [R2] Mijatovic, G., et al. (2021). An information-theoretic framework to measure the dynamic interaction between neural spike trains. IEEE Transactions on Biomedical Engineering, 68(12), 3471-3481.
   [R3] Shorten, D. P. et al. (2022). Early lock-in of structured and specialised information flows during neural development. Elife, 11, e74651.
   [R4] Mijatovic, G. et al. (2022). Measuring the rate of information exchange in point-process data with application to cardiovascular variability. Frontiers in Network Physiology, 1, 765332.

Response: We thank the reviewer for their referral to recent works exploring model-free analysis of causal interactions and synchronization in spike trains. As described in response to Reviewer 1's feedback, the manuscript has been revised to include a Related Work section under Methods that specifically examines the relation of the proposed method to existing methods, including a discussion of recent works on model-free approaches utilizing information theoretic approaches.

While the analyses of conditional independence based on transfer entropy in [R1, R3] do avoid assumptions imposed by binning the time axis, they test the conditional independence of the past of one point process and the updates of a second and hence more closely resemble tests of Granger Causality than synchrony. The mutual information rate approach in [R2, R4] do address synchrony explicitly by considering the transfer entropy between two point processes in both directions, but this measure remains restricted to a measure of pairwise interactions.

- *To address this comment of the reviewer, we have now added an entirely new section in the Methods, called "Related Work", which provides details and discussion of the existing relevant work, including the references pointed out by the reviewer (Pages 3-5).*

2) Since the mathematics behind the derivations is a bit heavy, it should be complemented by a more intuitive description of the features of the proposed methodology, where its properties are presented in layman terms; from this viewpoint, improving Fig. 1 and accompanying it with descriptive text would be useful.

Response: We thank the reviewer for their suggestions to improve the accessibility of the technical aspects of our work. In order to address these concerns, the presentation of our methodology in the main text has been substantially revised to only include essential mathematical details and provide greater consideration to intuitive descriptions of the proposed method's properties (Pages 6-8), while the comprehensive derivations have been moved to S1 Appendix.

3) Analysis parameters such as the data length, the bin width, and the length of the past window considered for history-dependent formulation of the method are crucial aspects in approaches to neural synchrony based on binning the temporal axis. To better understand the performance of the proposed methodology, its dependence on these parameters, as well as on their interplay, should be tested and discussed.

Response: We thank the reviewer for their suggestion to demonstrate the effect of analysis hyperparameters. We have now included new simulation results that address the effects of bin width and the length of the history integration window. Along with simulations previously present in S1 Appendix that addressed the choice of the forgetting factor, we have compiled these additional simulations in **S2 Appendix**. The effects of changing these hyperparameters is summarized briefly.

The bin width effectively defines the tolerance within which spike times are considered simultaneous – a large (small) bin width corresponds to a more relaxed (stringent) definition, hence allowing for more (less) higher-order simultaneous events. We show the spike and r-th order MkPP raster plots when simulated spiking responses are downsampled to mimic the effect of a larger bin size (See below, Fig. S1 reproduced from **S2 Appendix**).

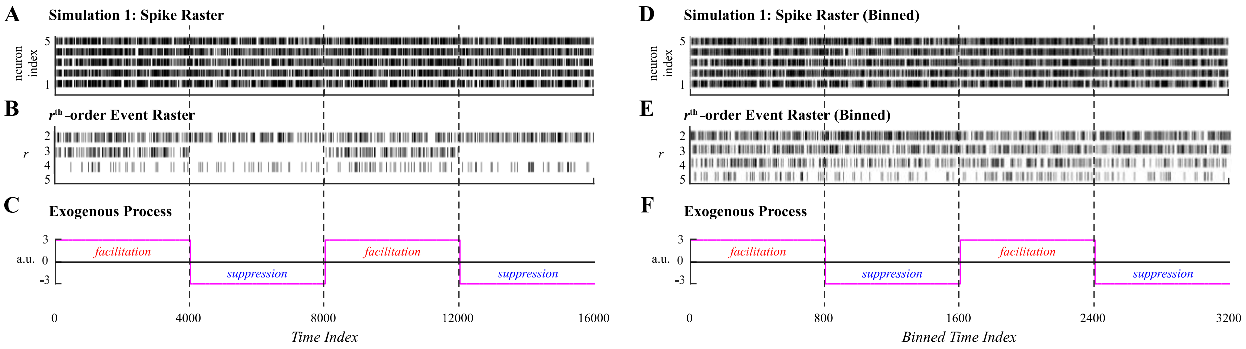

Fig S1. Large bin sizes results in loss of temporal resolution, distorting underlying correlational structure.

The MkPP rasters of the simulated data originally reflected dynamics of the simulated data; however, binning the spikes with bin width of 5 samples, it is apparent that the latent dynamics are not well reflected. In particular, frequent 5-th order events are observable after binning. This highlights that bin size constitutes an assumption about the relevant spike-timing precisions, and smaller bin sizes are preferrable when considering simultaneous spiking. In application to the human anesthesia and rat sleep datasets, bin widths were chosen to downsample the data length for tractability but while preserving the spike times – that is, bin width selections avoided instances that multiple spike times were binned together. This selection criterion is explicated stated in the revised text.

The length of recent ensemble spiking history are varied in additional analyses of simulated data that have been added to **S2 Appendix** (See below, Fig. S4 reproduced from **S2 Appendix**). The additional simulations show that the choosing the ensemble spiking history duration to be longer than the true duration does not drastically affect the statistical inference of higher-order spiking coordination (Fig. S4C), but shorter history duration (Fig. S4B) results in missing latent structure. Conservatively, a longer history dependence should be assumed, enabling greedy estimation to identify the most relevant covariates.

The dependence of the proposed method on the effective data length, $N_{eff} = \mathcal{O}(W/(1-\beta))$ had been characterized through simulation results presented in **S2 Appendix** (See below, Fig. S3 reproduced from **S2 Appendix**).

- *To address this comment of the reviewer, we have now added an entirely new appendix, namely **S2 Appendix**, containing several new numerical validations to demonstrate the effect of bin width and the history integration window, as well as the effect of varying W on model goodness-of-fit, that Reviewer 1 has recommended.*

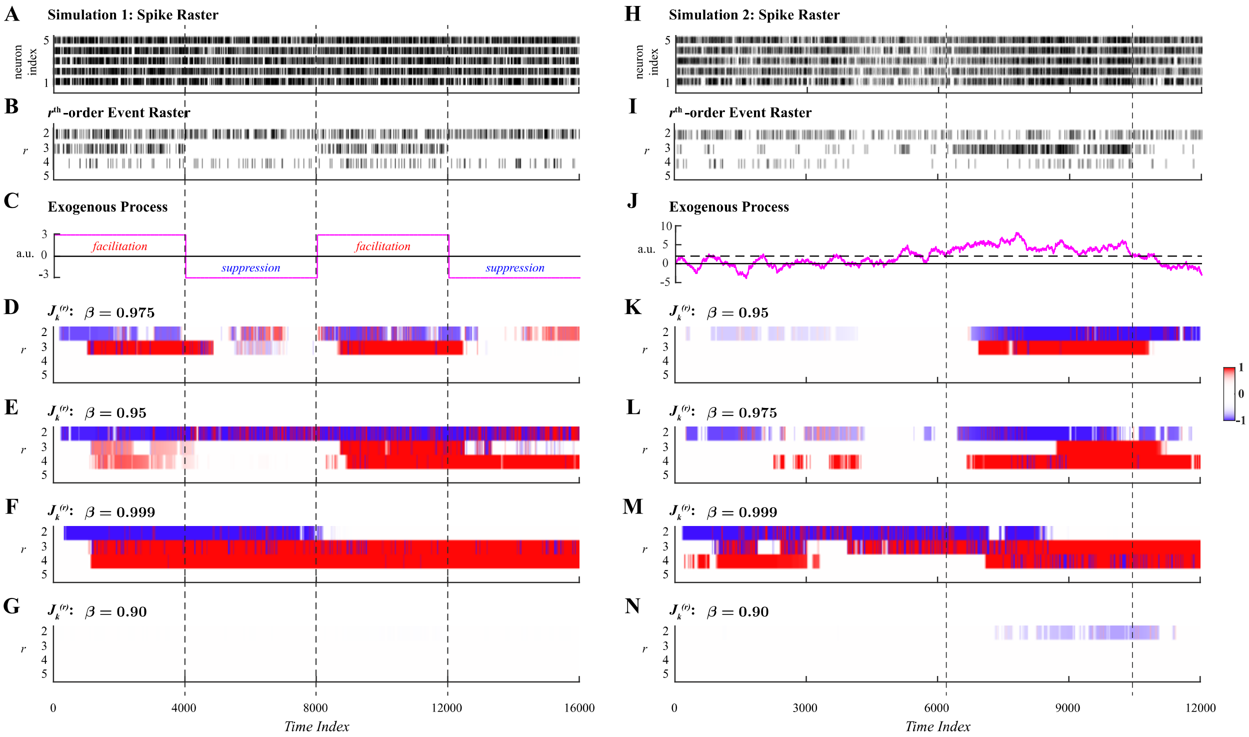

Fig S3. Effects of varying β in history-dependent analysis of simulated data.

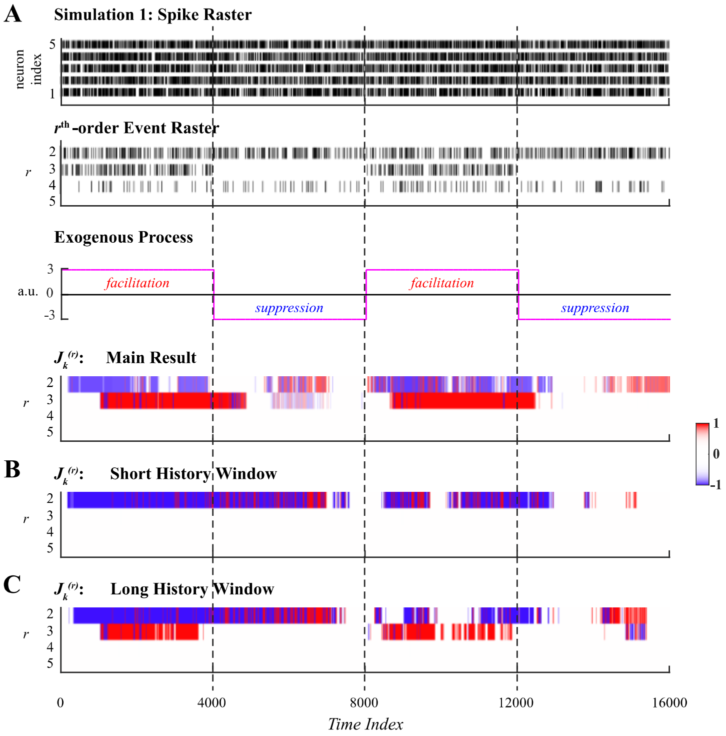

Fig S4. Effects of varying length of history-dependence on analysis of simulated data.
