# Supplementary material for: Adaptive modeling and inference of higher-order coordination in neuronal assemblies: A dynamic greedy estimation approach
Source: PLoS Comput Biol. 2024 May 28;20(5):e1011605. doi: 10.1371/journal.pcbi.1011605 (PMC11161120; doi:10.1371/journal.pcbi.1011605)
Supplement: Attachment — Submitted filename: MukherjeeBabadi_PLoSCompBiolR1_ReviewerResponses.pdf [file pcbi.1011605.s004.pdf]

Responses to Reviewer Comments on "Adaptive modeling and inference of higher-order coordination in neuronal assemblies: a dynamic greedy approach" (PCOMPBIOL-D-23-01661R1)

Shoutik Mukherjee and Behtash Babadi
Department of Electrical & Computer Engineering
Institute for Systems Research
University of Maryland, College Park, MD 20742, USA

Preface

We thank the reviewers and reviewing editors for their thoughtful feedback on our manuscript. We have now substantially revised the manuscript by including an additional simulation to address the scaling of the proposed method to large neuronal assemblies and evaluating the proposed method in comparison to dimensionality reduction analyses. In summary, the major changes include:

1) Revisions to the **Related Work** section that review PCA/ICA and compare them to the proposed methods.
2) The addition of a new simulation addressing the size of the neuronal assembly to **S2 Appendix** that analyzes spiking activity of 100 neurons.

In the following, we respond (in black) point-by-point to each of the reviewers' comments (in green). We have included identical versions of the main text and S2 Appendix that highlight changes (in red) for ease of tracking.

Response to the Comments of Reviewing Editor

The reviewers appreciated the attention to an important topic. Based on the reviews, we are likely to accept this manuscript for publication, providing that you modify the manuscript according to the review recommendations.

Unfortunately, we have had to recruit an additional reviewer at this stage because one of the original referees was unable to assess the revised manuscript. Both this new reviewer and myself feel that a little more work is needed to demonstrate the practical utility of this new method - first, by making some direct comparisons with established and widely used methods based on PCA or ICA, for example; and second, demonstrating that the method can be applied to larger scale data sets consisting of tens or hundreds of simultaneously recorded neurons, as are increasingly common in contemporary neuroscience.

Response: We thank the reviewing editor for their evaluation and suggestions to demonstrate the practical utility of our method. We have now compared our method to PCA/ICA and presented an application to an ensemble of 100 neurons. More details are given in response to the comments of Reviewer #3 below.

Response to the Comments of Reviewer #2

Reviewer #2:
The paper has been revised addressing all the concerns raised. In this reviewer opinion the paper deserves acceptance.

Response: We thank the reviewer again for their detailed suggestions that have improved the evaluation of the proposed methods and the accessibility of our manuscript to a broader readership.

Response to the Comments of Reviewer #3

Reviewer #3:
In this manuscript, the authors introduce a methodological framework that employs a discretized marked point process model with adaptive estimation techniques and statistical inference procedures to analyze coordinate ensemble activity. While the sophistication of the method allows for a nuanced representation of neural interactions, its complexity raises concerns regarding overfitting, generalizability, and statistical power. Furthermore, the mathematical presentation is densely packed and lacks intuitive explanations, which may limit accessibility to a broader audience. The framework demands a significant time investment for comprehension, potentially obscuring statistical limitations and impacting interpretability. Although complexity may be necessary for the proposed goals, the study does not convincingly demonstrate this necessity.

Response: We thank the reviewer for their suggestion to expand our demonstrations of the need for the proposed method's capability to capture nuances of ensemble spiking activity and address its scalability. We have outlined the revisions addressing the reviewer's specific comments below.

The necessity of such a complex framework is questioned by comparing it to existing, simpler methods known for detecting high-order correlations and co-activity patterns, such as those based on PCA or ICA (Chapin and Nicolelis 1999, Laubach et al. 1999, Peyrache et al. 2009, Lopes-dos-Santos et al. 2013). These approaches offer better interpretability and are widely used despite assumptions of linearity, non-stationarity, and being memoryless. The authors point out that their method does not suffer from these limitations but have not shown through simulations that it would outperform these simpler methods in practice due to these limitations. This comparison is crucial for justifying the new approach's complexity and provides a benchmark for

assessing the proposed method's incremental benefits, highlighting the importance of balancing sophistication with accessibility.

Response: We thank the reviewer for their referral to prior work that utilized PCA and ICA to detect latent sources of activity in ensemble spiking activity as a point of comparison for the proposed model. We note that these dimensionality reduction analyses assume that ensemble spiking is generated from a low-dimensional set of sources that are discovered based on empirical covariances. Consequently, for PCA, the recovered eigenmodes are reflective of groups of pairwise-correlated neurons, resembling the Pearson correlation analyses presented in Fig. 3 – Fig. 6 of the main text (see Fig. 3F below as an example is reproduced below). While ICA can identify sources that account for higher-order correlations, neither of these methods is well-suited to single-trial spike trains or when the underlying data structure is not low-dimensional or stationary. Moreover, they are unable to ascribe detected structure to endogenous or exogenous processes, a relevant consideration in the context of multi-region recordings, as one instance.

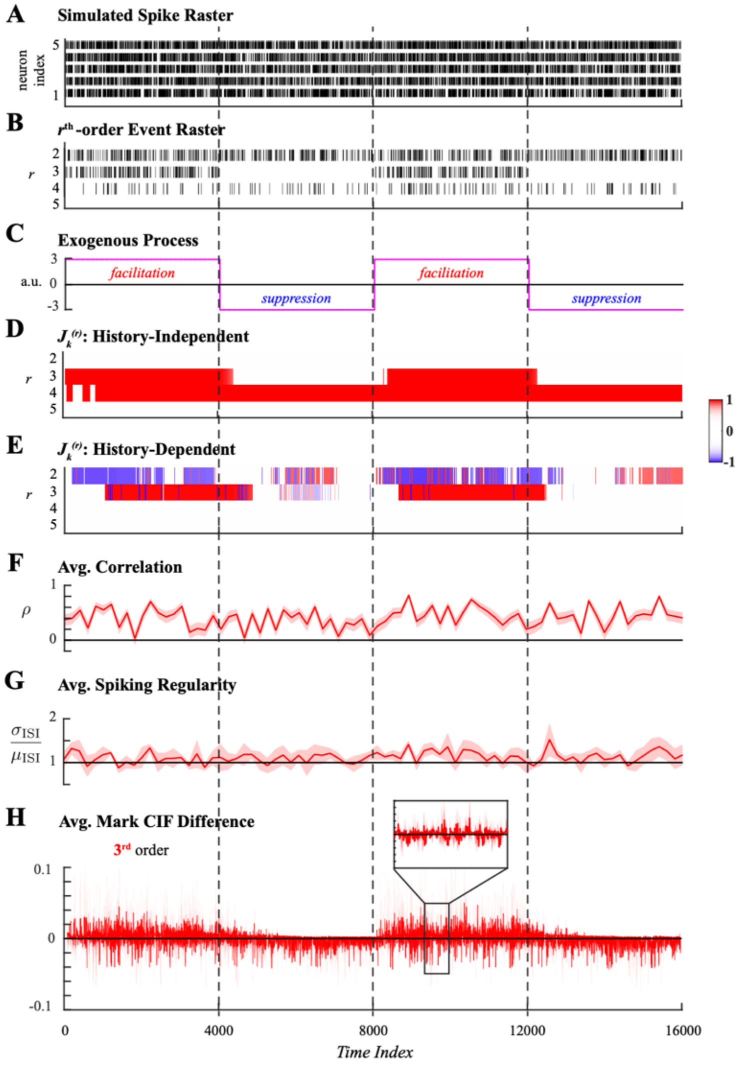

Figure 3: Simulated spiking activity of 5-neuron assembly with exogenously modulated 3rd-order interactions (A-C) analyzed using proposed history-independent, history-dependent analyses (respectively, D,E), average Pearson correlation (F), average spiking regularity (G), and the average instantaneous rate of 3rd-order events (H).

We applied the PCA and ICA approaches outlined in (Lopes-dos-Santos et al., 2013) to the simulated data set presented in Fig. 3, in which a square wave exogenously modulated 3rd-order simultaneous spiking events. The method first applies PCA to binned spike counts, then ICA to the significant eigenmodes (eigenvalues larger than 1) to identify latent sources of activity; in principle, this disentangles correlated but independent sources confounded in PCA. However, PCA failed to identify meaningful sources, as shown below. The 1st principal component (PC) indicates that all five neurons were correlated – namely that all pairs were correlated, as PCA is based on the empirical covariance – while the 2nd – 5th PCs all indicate groups of up to three pairwise (but weakly) correlated neurons. However, the PCs themselves bear little resemblance to any of the simulated data's latent dynamics. For completeness, we additionally performed ICA, which produced similarly uninterpretable components and are thus not shown.

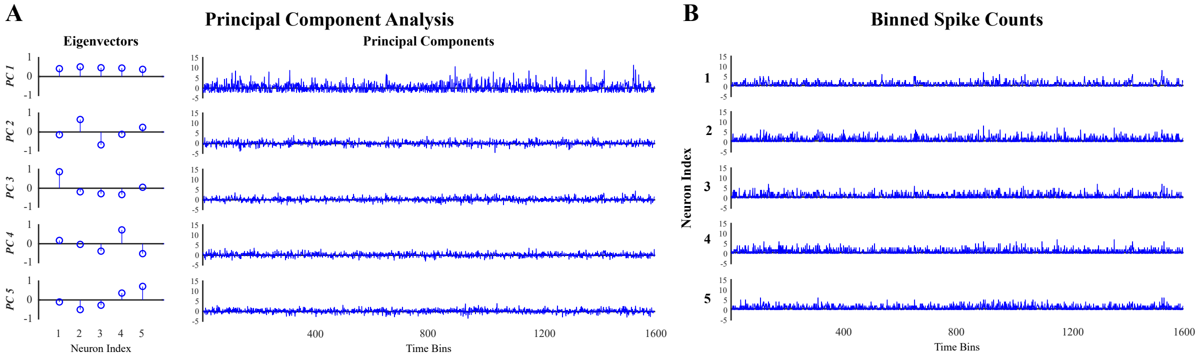

*Principal Component Analysis (**A**) of simulated data shown in Figure 3A—C. PCA was applied to the binned spike counts (**B**), utilizing the approach outlined in (Lopes-dos-Santos et al., 2013); bins were 10 frames long. The first principal component, like average Pearson correlation, indicates pairwise correlations between all pairs of neurons. However, neither the first nor the other principal components reflect the latent structure of the simulated spiking data.*

The result of PCA was hence similar to the Pearson correlation analysis shown in Fig. 3 in that the first principal component indicated strong pairwise correlations between all pairs of neurons, and did not recover the latent higher-order correlational structure of the assembly.

- *To address this comment of the reviewer, we have now augmented "Related Work" section of the Methods to include an evaluation of PCA/ICA and their relation to the proposed methods, including the references pointed out by the reviewer (Page 4). While PCA/ICA were applied to a simulated data set, their results were uninterpretable and hence excluded from the manuscript.*

Furthermore, the authors present their methodological advancements through the analysis of two simulated datasets, which, while illustrative of the method's potential, limit the demonstration of its versatility and robustness across a broader spectrum of neural coordination patterns. The simulations do not explore variations in coordination dynamics such as differing time scales, the size of neuronal assemblies, or the presence of multiple coordinating assemblies. Moreover, the choice to simulate the activity of only five neurons raises concerns about the method's scalability and its ability to maintain statistical power when faced with the increased dimensionality typical of

larger neuronal ensembles. This limitation gives the impression that the method may not be well-suited to handle the complexity of real-world neural data, where the number of neurons can be significantly larger, without succumbing to the curse of dimensionality. Addressing these concerns by incorporating a wider range of simulation scenarios and offering a detailed comparative analysis with established methods would significantly strengthen the manuscript, enhancing the credibility and perceived utility of the proposed methods within the neuroscience research community.

Response: We thank the reviewer for their suggestion to expand our simulations to consider the size of neuronal assemblies. In order to address this concern, we have added a new simulated example to **S2 Appendix**, presented in Figure S5 which is reproduced here. In this new simulation, the spiking activity of 100 neurons was generated so that there was no exogenous coordination or significant synchronization in the first half of the simulated duration, while in the second half, 3rd-order spiking events (specifically, a subset of all 3rd-order interactions) were exogenously coordinated. The mark space, consisting of 2^{100}-1 marks, was reduced to the set of reliable interactions, consisting of all 100 1st-order events, all 4950 2nd-order events, and 50 frequently occurring 3rd-order events; the set of reliable interactions thus consisted of 5100 events. The proposed method was used to model dynamics in the rates of reliable interactions; as shown in Figure S5C (reproduced below), the proposed inference procedure accurately captured the latent dynamics in higher-order coordination of ensemble spiking.

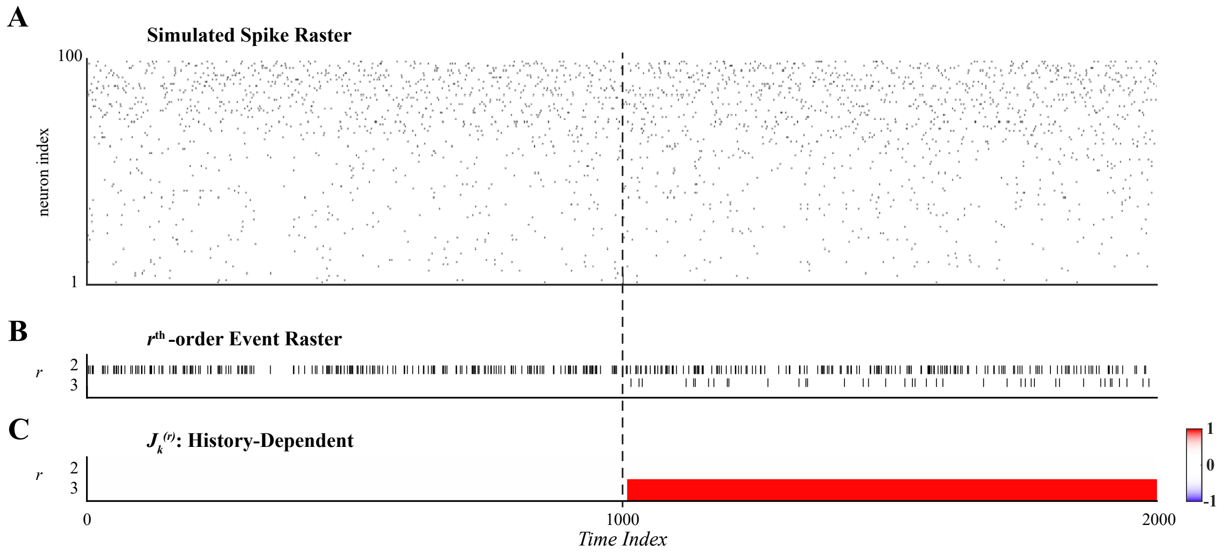

Figure S5: History-dependent analysis of higher-order coordinated spiking in assembly of 100 neurons. ***A.*** *Simulated spiking activity of 100 neurons with no exogenous coordination for the first half of the simulated duration and exogenously induced 3rd-order interactions during second half of simulated duration.* ***B.*** *Latent dynamics of higher-order spiking were evident in raster of rth-order spiking events.* ***C.*** *Exogenous coordination was correctly detected using the history-dependent model of the marked point process.*

- *To address this comment of the reviewer, we have now added a new simulation to* ***S2 Appendix****, presented in Figure S5, that employs the proposed method to analyze the ensemble spiking activity of 100 neurons.*

For the real data application, the proposed analytical methods to a brief 182-second sleep cycle, analyzing the activity of only ten neurons from each class (pE and pI), again raises concerns about the method's computational demands and scalability. This limited scope suggests that the method may be computationally intensive, potentially restricting its practicality for studies requiring long-term or large-scale neural recordings. Addressing the computational efficiency and scalability of the method is crucial, especially in neuroscience research where understanding complex neural networks often involves analyzing data from dozens to hundreds of neurons over longer periods. Without addressing these concerns, the method's potential impact and adoption within the neuroscience community could be significantly limited.

Response: We agree with the reviewer that scalability is a crucial consideration. Key aspects of the proposed method (namely: greedy estimation, which involved efficient recursive gradient and log-likelihood computations; and pruning the space of all events to focus on reliable interactions) were motivated by the need to address the tractability of an inherently high-dimensional problem. Moreover, the results presented in our manuscript are based on analyses that are intentionally conservative in reducing the dimensionality of the problem (namely, in reducing the mark space). While this demonstrates the advantages to detailed modeling of ensemble spiking activity, we also have noted that the proposed framework generalizes for less computationally demanding problems (e.g. modeling at most 3rd-order interactions).

Regarding the reviewer’s specific concern about tractability in relation to spiking activity during a 182-second sleep cycle, we first note that the data set consisted of only 10 pI neurons, so for parity 10 pE neurons were selected for comparison. Moreover, the complexity of the proposed method is due to the dimensionality of the mark space rather than the duration of the data. The 100-neuron simulation example presented in response to the reviewer’s previous comments on the simulated examples addresses the principle cause for concern about the tractability of the proposed method.

- *To address this comment of the reviewer, we have now added a new simulation to* ***S2 Appendix****, presented in Figure S5, that employs the proposed method to analyze the ensemble spiking activity of 100 neurons.*
